# Tracking Fungal Growth: Establishment of Arp1 as a Marker for Polarity Establishment and Active Hyphal Growth in Filamentous Ascomycetes

**DOI:** 10.3390/jof7070580

**Published:** 2021-07-20

**Authors:** Anika Groth, Carolin Schunke, Eva Johanna Reschka, Stefanie Pöggeler, Daniela Elisabeth Nordzieke

**Affiliations:** 1 Institute of Microbiology and Genetics, Genetics of Eukaryotic Microorganisms, Georg-August-University of Göttingen, Grisebachstr. 8, 37077 Göttingen, Germany; anika.gibron@stud.uni-goettingen.de (A.G.); carolin.schunke@bzh.uni-heidelberg.de (C.S.); evareschka@gmx.net (E.J.R.); 2 Biochemistry Center, Heidelberg University, Im Neuenheimer Feld 328, 69120 Heidelberg, Germany

**Keywords:** Arp1, polarity, microscopy, *Sordaria macrospora*, *Colletotrichum graminicola*, chemotropism

## Abstract

Polar growth is a key characteristic of all filamentous fungi. It allows these eukaryotes to not only effectively explore organic matter but also interact within its own colony, mating partners, and hosts. Therefore, a detailed understanding of the dynamics in polar growth establishment and maintenance is crucial for several fields of fungal research. We developed a new marker protein, the actin-related protein 1 (Arp1) fused to red and green fluorescent proteins, which allows for the tracking of polar axis establishment and active hyphal growth in microscopy approaches. To exclude a probable redundancy with known polarity markers, we compared the localizations of the Spitzenkörper (SPK) and Arp1 using an FM4-64 staining approach. As we show in applications with the coprophilous fungus *Sordaria macrospora* and the hemibiotrophic plant pathogen *Colletotrichum graminicola*, the monitoring of Arp1 can be used for detailed studies of hyphal growth dynamics and ascospore germination, the interpretation of chemotropic growth processes, and the tracking of elongating penetration pegs into plant material. Since the Arp1 marker showed the same dynamics in both fungi tested, we believe this marker can be broadly applied in fungal research to study the manifold polar growth processes determining fungal life.

## 1. Introduction

Polar tip growth as a form of cell extension is widespread in eukaryotes and can be found in neurons, root hairs, pollen tubes, and at the apex of fungal hyphae [1,2]. In fungi, polar growth allows for not only radial growth but also hyphal network formation by branching followed by the targeted fusion of fungal cells [3,4]. Furthermore, the apical growth direction can be adapted by the sensing of light, physical contact, electrical currents, and chemicals, including nutrients, pheromones, and host-derived signals [5,6]. For plant-interacting symbionts and pathogens, the penetration peg formation and elongation outgoing from appressoria, hyphopodia (or so-called expressoria recently identified in *Epichloë festucae*) requires strict polar growth [7,8,9,10,11].

Before apical growth can start, cell polarity that enables the asymmetrical transport of cellular components for cell wall and plasma membrane extension must be established [1,12,13,14]. The recruitment of Rho-GTPase Cdc42 to the new polarity site is crucial for this process [13,15,16,17,18]. After Cdc42 accumulation, factors such as the septins and homologs of the yeast formin Bni1 are located on the new polar axis followed by the formation of actin or septin rings, respectively [19,20,21,22,23,24]. As work on *Neurospora crassa* has shown, a second Rho-GTPase, Rac1, is recruited to the polar site and regulates chemotropic growth processes, including germling and hyphal fusion [25]. A prominent molecular structure correlated to hyphal polarity is the Spitzenkörper (SPK), a dark-phase structure close to the hyphal tip. Actin cables and microtubules (MTs), which are surrounded by vesicles either bound for fusion with the plasma membrane at the hyphal apex or endocytic vesicles involved in the recycling of membranes and proteins, are central to the SPK [11,13,26,27,28].

At a polar growing hypha, outward- and inward-bound traffic meet. This requires specific transportation systems for the coordination of stable polar growth [29]. Though actin and their motor protein myosin mediate short-distance transport from the SPK to the plasma membrane, long distance transport to the SPK takes places along MTs [1]. In filamentous fungi, MTs are polar structures that show a bidirectional polarity inside mature hyphae and clear plus-end polarity in the apical region [30,31,32]. Conserved among the eukaryotic kingdoms of life, the minus-end directed transport along MTs is dependent on the interaction of the multisubunit complexes dynein and dynactin [33]. Though dynein is the motor sitting on the MTs, different cargos are linked to the motor by dynactin. Dynactin is composed of a projecting sidearm, which interacts with the dynein motor, and a 37 nm minifilament, which binds to the cargo [33,34]. Eight monomers of actin-related protein 1, Arp1, the protein of its class that is able to form filaments, are central to the minifilament [35,36,37]. As was shown for *Ustilago maydis* and *Aspergillus nidulans*, cytoplasmic dynein is recruited to the plus-ends of MTs in a kinesin-dependent manner, resulting in the formation of a dynein loading zone close to the hyphal tip [38,39,40,41]. Together, dynein and dynactin allow for endoplasmic reticulum (ER)-to-Golgi vesicle trafficking, the minus-end directed transport of organelles, late and early endosomes, protein complexes, and lipid droplets, as well as the centripetal movement of virus capsids and ER–Golgi transport complexes [32,33,34,42,43].

In our study, we investigated the localization dynamics of the dominant dynactin component Arp1 using protein fusion constructs with the red and green fluorescence tags of TagRFP-T and mNeonGreen (mNG), respectively. As the monitoring of Arp1 in the coprophilous fungus *Sordaria macrospora* and the hemibiotrophic plant pathogen *Colletotrichum graminicola* has shown, Arp1 fusion proteins localize to tips of expanding hyphae and the sites of polar growth establishment, whereas they are absent from non-growing hyphae and future germling fusion sites. Furthermore, dynamic dot-like localization close to nuclei has indicated a role in nuclear transport and sorting. To test for the probable applicability of fluorescent Arp1 as a polarity marker, we monitored its localization during several polar growth processes, such as vegetative polar growth, ascospore germination, chemotropic growth, and penetration hyphae elongation. We additionally compared the localization of Arp1 and the SPK by co-staining with FM4-64. As our results showed, the usage of the Arp1 marker protein allows for dynamic growth processes to be followed in saprophytic and pathogenic fungal species, and it enables an improved assessment of chemotropic growth analyses. Since the Arp1 marker protein has overlapping but not identical localization characteristics compared with the SPK, we believe it will be a valuable extension to existing marker proteins.

## 2. Materials and Methods

### 2.1. Strains, Media and Growth Conditions

For the cloning and propagation of recombinant plasmids, *Escherichia coli* strain MACH1 (Thermo Fisher Scientific, Waltham, MA, USA) was used in standard culture conditions [44]. To generate recombinant plasmids via homologous recombination in yeast, positive transformants of *Saccharomyces cerevisiae* strain PJ69-4A were selected for uracil prototrophy [45,46].

*S. macrospora* wild-type strain DSM977 was transformed with recombinant plasmids according to the standard protocol [47]. Positive transformants were selected on media containing nourseothricin-dihydrogen sulphate (50 µg/mL, nat) (Jena Bioscience GmBH, Jena, Germany) and/or hygromycin B (110 U/mL, hyg) (Merck, Kenilworth, NJ, USA). *S. macrospora* strains were grown on liquid or solid biomalt maize medium (BMM) under continuous light conditions at 27 °C [48,49,50]. Crosses of *S. macrospora* strains were performed as described previously [51]. After incubation for 7–10 d, plates were topped with an agar-plate containing nourseothricin and hygromycin B to select positive single-spore isolates.

The wild-type CgM2 strain (M1.001) of *C. graminicola* (Ces.) G. W. Wilson (teleomorph *Glomerella graminicola*) was used in this study [52]. For the generation of falcate conidia, *C. graminicola* was grown on oat meal agar (OMA) for 14–21 d at 23 °C [10]. Oval conidia, used as the basis for *C. graminicola* transformation and experimental procedures, were obtained in shaking cultures in a liquid complete medium with 0.5 M of sucrose (CMS) for two days (80 rpm and 23 °C), followed by 5–8 days of incubation in darkness [53]. For the selection of successful transformants and growth analyses, a complete medium (CM) containing nourseothricin-dihydrogen sulphate (150 µg/mL, nat; Jena Bioscience GmBH, Jena, Germany) or geneticin disulphate (G418, 400 µg/mL, gen; Carl Roth GmbH & Co. KG, Karlsruhe, Germany) was used when appropriate.

Details about all used strains are given in Table 1.

### 2.2. Multiple Sequence Alignment of Fungal Arp1

Multiple sequence alignments of protein sequences and neighbor joining phylogenetic analysis were performed with MAFFT version 7 [54]. A bootstrap analysis was conducted with 1000 iterations to test the tree for statistical significance. The tree was displayed with Archaeopteryx.js [55].

### 2.3. Construction of Plasmids

Plasmids were generated via homologous recombination in *S. cerevisiae* [45], as described previously [56], or the NEBuilder HiFi DNA Assembly Cloning Kit (New England Biolabs, Ipswich, MA, USA) according to the instruction manual. Information on all primer sequences and plasmids is provided in Tables S1 and S2, respectively.

For the construction of the pArp1-KO knockout plasmid, the Golden Gate cloning system according to Dahlmann et al., 2020, was used [57]. The 5′-(1311 bp) and 3′-(1791 bp) flanking regions of the *Smarp1* gene were amplified from *S. macrospora* wt gDNA with the Arp1-ko-5f/Arp1-ko-5r and Arp1-ko-3f/Arp1-ko-3r primer pairs, respectively. Together with the donor vector pGG-hph and the destination vector pDest-Amp, the fragments were cloned via the Golden Gate procedure [57].

To generate the pSmarp1 plasmid, a fragment (5028 bp) containing the native promotor, coding sequence and native terminator of *Smarp1* was amplified from *S. macrospora* wt gDNA with the Arp1-nat-5f/Arp1-nat-3r primer pair. The fragment was cloned into the *Xho*I-linearized pRS-nat vector [58] via homologous recombination using the PJ69-4A yeast strain [45].

The fragment containing the native promoter and coding sequence of *Smarp1* was amplified from *S. macrospora* wt gDNA using the Arp1_promotor_f/Arp1-Neo_r primer pair for the generation of the pSmarp1-mNG plasmid. The amplification of *mNG* was done with the Neo_f/Neo_TtrpC_r primer combination using the pmn-xyl plasmid [59]. The terminator of the anthranilate synthase gene *trpC* of *Aspergillus nidulans*, *TtrpC*, was amplified from the p1783-1 plasmid [60] with the TtrpC_f/TtrpC_pRS_r primer pair. The three PCR products were cloned into *Xho*I-linearized vector pRS-nat [58] via homologous recombination in the PJ69-4A yeast strain [45]. To construct the pSmarp1-TagRFP-T_nat/_hyg plasmids, the *Smarp1* promoter and coding sequence was amplified with the Arp1_5′f/Arp1_RFP_r primer pair from *S. macrospora* wt gDNA. The TagRFP-T sequence and the *A. nidulans TtrpC* were amplified using the primer pairs of RFP-f/RFP-r-trpC from pTagRFP-T_nat [61] and TrpC_F/pRS426GFPrev from p1783-1 [60], respectively. The *S. cerevisiae* strain PJ69-4A was transformed with the three amplicons and *Xho*I-linearized pRS-hyg [62] or pRS-nat [58] to generate recombinant plasmids via homologous recombination [45,46].

For the generation of the pCgarp1-TagRFP-T plasmid, three fragments were amplified for the assembly with pJet1.2 (Thermo Fisher Scientific, Waltham, MA, USA) using the NEBuilder HiFi DNA Assembly Cloning Kit (New England Biolabs). *Cgarp1*, including the 1 kb 5′ region, was amplified from CgM2 gDNA using the 5′CgArp1_pJet_fw/CgArp1_rev oligonucleotides. The coding sequence of TagRFP-T, including the *TtrpC* terminator of *A. nidulans*, was obtained from pSmarp1-TagRFP-T (TagRFP-T_CgArp1_fw/TtrpC_r). The neomycin phosphotransferase *nptII* resistance cassette, was amplified from the pII99 plasmid [63] using nptII_TtrpC_fw/PtrpC_pJet_rev. For further subcloning, *EcoR*V restriction sites were integrated in the oligonucleotide sequences of 5′CgArp1_pJet_fw and nptII_TtrpC_fw. Outgoing from pCgarp1-TagRFP-T, the pJet_gen plasmid harboring the *nptII*-resistance cassette was generated via hydrolysis with *EcoR*V and subsequent ligation of the plasmid backbone.

Primers were synthesized by Sigma-Aldrich Chemie GmbH (Taufkirchen, Germany). The DNA sequencing of the plasmids was performed by Seqlab Sequence Service Laboratories GmbH (Göttingen, Germany).

### 2.4. Generation of the Smarp1 Deletion Strain ∆Smarp1

To delete the *S. macrospora arp1* gene, the pArp1-KO knockout plasmid was used as a template to amplify the 4568 bp deletion cassette with the GG_KO_fw/GG_KO_rv primer pair containing the 5′- and 3′- flanking regions of *Smarp1* and the *hph* cassette. After the PCR clean-up of the amplicon, the *S. macrospora* ∆Smku70 strain was transformed with the deletion cassette to replace the *Smarp1* ORF with the *hph*-cassette. Primary transformants were crossed with the spore-color mutant fus1-1 (S23442) [64], and single-spore isolates (ssi) of ∆Smarp1 carrying hygromycin resistance were selected [51]. The verification of the absence of the *Smarp1* gene and the integration of the *hph*-cassette at the desired locus were performed with the Arp1-v5f/h3 (1578 bp) and Arp1-v5f/Arp1-vORF5-r (2170 bp) primer pairs, respectively. To check the presence of the *Smku70* gene in ∆Smarp1 after crossing, the Smku70-v1-f/ku70-ko-v3f(R) (2851 bp) primer pair was used. The deletion of ∆Smarp1 was verified via Southern hybridization hydrolyzing the gDNA of *S. macrospora* wt, ∆Smku70, and ∆Smarp1 with *EcoR*V. After electrophoresis, a capillary blot with a nylon membrane was performed at RT overnight. The 1791-bp 3′-probe was amplified from *S. macrospora* wt gDNA with the Arp1-ko-3f/Arp1-ko-3r primer pair. The labeling of probes for Southern blot experiments was done using the Amersham AlkPhos Direct Labelling and Detection Kit (GE Healthcare, Amersham RPN3680, Boston, MA, USA). Detection was performed according to the manufacturer’s manual. Signals were visualized on X-ray films (Amersham Hyperfilm TM ECL; GE Healthcare, Boston, MA, USA) using an “Optimax X-ray film processor” (PROTEC GmbH & Co. KG, Oberstenfeld, Germany).

The complementation of ∆Smarp1 was performed by transformation with the pSmarp1-TagRFP-T_nat plasmid or its untagged Arp1 version, pSmarp1, resulting in the ∆Smarp1::Smarp1-TagRFP-T or ∆Smarp1::Smarp1 strains, respectively. Positive primary transformants were selected on BMM supplemented with nourseothricin and hygromycin. Crosses were performed as described previously [51].

**Table 1:** Strains used in this study.

Name of Strain	Genotype	Reference
*Sordaria macrospora*		
DSM997	*S. macrospora* wild-type (wt)	DSMZ
S23442	mutation in *fus1-1* gene, brownish ascospores, fertile	[64]
fus::RH2B	ectopic integration of pRH2B into S23442; hyg^R^, pt, *Pgpd::hh2b::tdTomato::TtrpC*	[65]
wt::TagRFP-T	ectopic integration of ptRFP_nat into DSM997; nat^R^, ssi, *Pccg1::TagRFP-T::TtrpC*	[61]
wt::GG-C-F-mNG	ectopic integration of pGG-C-F-mNG in DSM997; nat^R^, ssi, *Pgpd::mNG::3xFLAG::TtrpC*	[59]
∆Smku70	∆Smku70::nat^R^, fertile	[66]
Sm::Smarp1-TagRFP-T	ectopic integration of pSmarp1-TagRFP-T in DSM997; hyg^R^, ssi, *Smarp1P::Smarp1::TagRFP-T::TtrpC*	this study
Sm::Smarp1-mNG	ectopic integration of pSmarp1-mNG in DSM997; nat^R^, ssi, *Smarp1P::Smarp1::mNG::TtrpC*	this study
∆Smarp1	∆Smarp1::hyg^R^, sterile	this study
∆Smarp1::Smarp1	ectopic integration of pSmarp1 in ∆Smarp1; hyg^R^, nat^R^, ssi, *Smarp1P::Smarp1::Smarp1T*	this study
∆Smarp1::Smarp1-TagRFP-T	ectopic integration of pSmarp1-TagRFP-T in ∆Smarp1; hyg^R^, nat^R^, ssi, *Smarp1P::Smarp1::TagRFP-T::TtrpC*	this study
*Colletotrichum graminicola*		
CgM2	*C. graminicola* wild-type (wt); also referred to as M1.001	[67]
CgM2::Smarp1-mNG	ectopic integration of pSmarp1-mNG in CgM2; nat^R^, ssi, *Smarp1P::Smarp1::mNG::TtrpC*	this study
CgM2::Cgarp1-TagRFP-T	ectopic integration of pCgarp1-TagRFP-T in CgM2; gen^R^, ssi, *Cgarp1P::Cgarp1::TagRFP-T::TtrpC*	this study

nat^R^: nourseothricin resistant; hyg^R^: hygromycin resistant; gen^R^: geneticin disulphate resistant; ssi: single-spore isolate; pt: primary transformant; *P*: promotor region; *TtrpC*: terminator of the anthranilate synthase gene of *A. nidulans*; *Pccg1*: promoter of the *clock controlled gene 1* of *N. crassa*; *Pgpd*: promoter of the glyceraldehyde-3-phosphate dehydrogenase gene of *A. nidulans*; *mNG*: gene for green fluorescence protein monomeric NeonGreen (mNG) of *Branchiostoma lanceolatum*; *tdTomato*: gene encoding red fluorescence protein tdTomato from *Discosoma* species; *TagRFP-T*: gene for red fluorescence protein TagRFP-T of *Entacmaea quadricolor*; *hh2b*: gene for histone 2b.

### 2.5. Generation of S. macrospora Strains for Fluorescence Microscopy

Strains were generated by transforming wt *S. macrospora*, as described previously [47]. The ectopic integration of the pSmarp1-TagRFP-T and pSmarp1-mNG plasmids led to the selection of positive transformants on BMM supplemented with nourseothricin-dihydrogen sulphate (50 µg/mL) and/or hygromycin B (110 U/mL) (Merck, Kenilworth, NJ, USA). Primary transformants were grown over 7–9 d at 27 °C to obtain single-spore isolates, as described previously [51].

### 2.6. Generation of C. graminicola Strains

The pCgarp1-TagRFP-T and pSmarp1-mNG plasmids were linearized using *Not*I and *Nde*I, respectively, prior to transformation in CgM2. Protoplasts were obtained from oval conidia by cell wall digestion using a lysis enzyme of *Trichoderma harzianum,* as described previously [10]. To obtain homokaryotic transformants, colonies that had developed on CM plates supplemented with 400 µg/mL geneticin disulphate (pCgarp1-TagRFP-T) or 150 µg/mL nourseothricin-dihydrogen sulphate (pSmarp1-mNG) were allowed to conidiate on OMA. After single-spore isolation using the generated falcate conidia, resistant transformants were tested for the expression of the red or green fluorescent Arp1 fusion proteins by fluorescence microscopy.

### 2.7. Fluorescence Microscopy

Microscopic documentation was performed with the AxioImager M1 microscope (Zeiss, Jena, Germany) with differential interference contrast (DIC) and specific filter sets for the detection of the fluorescent signals. Image capturing was performed with a Photometrix CoolSNAP HQ camera (Roper Scientific, Photometrics, Tucson, AZ, USA), and images were processed using ZEISS ZEN Digital Imaging (version 2.3; Zeiss). To detect the mNG signal, a 49002 Chroma filter set (exciter ET470/40x, ET525/50m, beam splitter T495lpxr) was used, and for tdTomato/TagRFP-T/FM4-64 signals, a 49005 Chroma filter set (exciter ET545/30x, emitter ET620/60 m and beam splitter T570LP) was used. For each experiment, at least three biological replicates were analyzed more than three times.

For the investigation of *S. macrospora* hyphae, the strains were grown for 24 h on BMM-covered glass slides and 2 mL of liquid BMM on top under continuous light at 27 °C. For FM4-64 (Thermo Fisher Scientific, Waltham, MA, USA) staining, the Sm::Smarp1-mNG strain was grown on solid BMM supplemented with 1.5% agarose (Biozym Scientific GmbH, Hessisch Oldendorf, Germany) at 27 °C under continuous light conditions for 24 h. Then, 100 µL of an FM4-64 solution (1 µg/mL in distilled water) was applied to the mycelium on the agar-piece and incubated for 15 min at 37 °C. For nuclei staining, DAPI (AppliChem, Darmstadt, Germany) was dissolved in distilled water to a stock solution of 1 mg/mL and further diluted with methanol to a concentration of 1 µg/mL. Incubation took place in the dark at 37 °C for 2–3 min. For time lapse studies of growing hyphae, *S. macrospora* strains were grown on BMM or Sordaria Westergaard (SWG) fructification medium [48,49,50] supplemented with 1.5% agarose (Biozym Scientific GmbH, Hessisch Oldendorf, Germany) under continuous light conditions for 24 h at 27 °C. For the analysis of SmArp1-mNG localization during ascospore germination, the wt::Smarp1-mNG x fus::RH2B strain was grown on BMM over 7–9 d, and discarded spores were washed down with 0.02% Tween 20. Ascospores were then plated on BMM containing 1.5% agarose, and pieces were cut out and analyzed under the fluorescence microscope after 3–4 h. Recording interval of 5 s over 20 min were used for time lapse studies. To analyze Arp1-mNG localization at nuclei, the ratio of Arp1-mNG localization close to a nucleus to the total number of nuclei was estimated for the 36 single pictures, thus resulting in Video S2.

The recording of *C. graminicola* hyphae expressing the Arp1 of *S. macrospora* C-terminally fused to mNG was performed after incubation for 3 d at 23 °C on CM covered with cellophane. The staining of these hyphae with FM4-64 (Thermo Fisher Scientific, Waltham, MA, USA) was done by the application of 100 µL of a 1 µg/mL working solution on the growing mycelium, followed by incubation for 15 min at 37 °C. For the analysis of Cgarp1-TagRFP-T localization during early germination and germling fusion, 50 µL of oval conidia (c = 5 × 10^7^/mL) were spread on water agar (1% agarose, 1% Serva agar, and 25 mM NaNO_3_). Incubation took place for 2 h (monitoring of young germlings) or 6 h (monitoring of CAT fusions and older germlings) at 23 °C. For time lapse studies of young germlings, a recording interval of 5 min was used. To track Cgarp1-TagRFP-T localization during plant penetration, a heat-inactivated onion epidermis covering water agar (1% agarose and 1% Serva agar) was inoculated with 10 µL drops containing 10^3^ oval conidia in 0.01% Tween for 29 h at 23 °C [68]. Different layers of an infected onion epidermis were recorded at a fixed distance of 0.5 µm. All experiments were performed with a minimum of three independent replicates.

### 2.8. Analysis of Chemotropic Growth Using the Arp1-TagRFP-T Marker

The application of Cgarp1-TagRFP-T for the analysis of chemotropic growth was performed by confronting germlings derived from *C. graminicola* oval conidia (c = 5 × 10^5^/mL) with a growing gradient of 50 mM glucose using a 3D-printed device, as described previously [69]. After 6 h of incubation at 23 °C with a minimum of 40 germlings/experiment, whether the tips of fungal germlings were growing to the established glucose gradient or to the solvent control (water) was determined. To allow for the discrimination of active growing germlings and to include non-germinated conidia with an established polar axis in the analysis, the localization of the Arp1-TagRFP-T maker was also tracked. In the final analysis, only germlings and conidia with a clear Arp1-TagRFP-T localization at the tip or a distinct cellular site were evaluated, respectively. In the case that one germling showed Arp1-TagRFP-T localization at two tips, only individuals with a preferred growth direction were included into the analysis. From the obtained numbers, the chemotropic growth rate was calculated as described previously [69]. All experiments were performed with a minimum of five independent replicates. For statistical analyses, the t-test for equal variances was used for all displayed experiments.

### 2.9. Protein Sample Preparation and Western Blot Hybridization

*S. macrospora* strains were cultivated in liquid BMM and grown for 3 d at 27 °C to extract proteins from fungal mycelium. After harvesting, drying, and grinding the mycelium in liquid nitrogen, 520 µL of lysis buffer (10 mM Tris-HCl pH 7.5, 150 mM NaCl, 0.5 mM EDTA pH 8.0, 1 mM PMSF, 2 mM DTT, 0.5% NP-40, 1× protease inhibitor cocktail IV (1 tbl/50 mL, 04693132001, Mannheim, Germany), and 1× PhosSTOP™ (1 tbl/10 mL, Roche, Mannheim, Germany))/g mycelium powder were added. Together with about 200 µL of glass beads (Ø 0.25–0.5 mm; Roth GmbH, Karlsruhe, Germany), cells were lysed in the TissueLyser (Qiagen, Hilden, Germany) at 30 Hrz for 2 min. Subsequently, cells were separated from debris by centrifugation at 10,000 rpm for 15 min at 4 °C and were prepared for Western Blot analysis by adding 4× NuPAGE^®^ LDS-SB (Thermo Fisher Scientific, Waltham, MA, USA) and 1 M DTT to the crude extract. After heating of the samples for 10 min at 70 °C, 25 µL were loaded on a 12% SDS gel. As a protein standard, the Nippon Genetics Co. Europe blue star pre-stained protein marker (NIPPON Genetics Europe, Düren, Germany) was used.

Blotting was performed using the AmershamTM ProtranTM Nitrocellulose Blotting Membrane (GE Healthcare, Little Chalfont, UK) with 1× transfer buffer and a Mini Trans-Blot^®^ Cell device, as described by the manufacturer (Bio-Rad Laboratories, Hercules, CA, USA) [70]. The nitrocellulose membrane was blocked with 5% (*w*/*v*) skim milk powder in 1× Tris-buffered saline supplemented with 0.05% Tween 20^®^ (TBST) for 1 h at RT. To detect antigen–antibody reactions, a primary TagRFP-T (rabbit) -antibody (1:12,500, BioCat (Evrogen, Moscow, Russia), AB233-ev) solved in 5% skim milk/TBST was used. After removing the primary antibody, the membrane was washed three times with 1× TBST for 15 min, and a horse-radish peroxidase (HRP)-coupled secondary anti rabbit-antibody (1:5000, Thermo Fisher Scientific, Waltham, MA, USA) was applied to the membrane for 1 h at RT. To detect the HRP-coupled antibodies, the ImmobilonTM Western HRP Substrate kit (Merck, Kenilworth, NJ, USA) was used. Signals were visualized on X-ray films (Amersham Hyperfilm TM ECL, Marlborough, MA, USA) using an “Optimax X-ray film processor” (PROTEC GmbH & Co. KG, Oberstenfeld, Germany).

### 2.10. Phenotypic Analysis

To define growth behavior and sexual development, *S. macrospora* strains were grown on solid SWG medium over 10 d and documented with a VHX-500F Digital Microscope (Keyence, Osaka, Japan). For growth-rate determination, six replicates each of *S. macrospora* wt strain, Sm::Smarp1-TagRFP-T and Sm::Smarp1-mNG, were grown in 30-cm race tubes filled with solid SWG medium over 7 d under continuous light at 27 °C. After 3 d, the growth front was marked every day at the same time to determine the growth velocity in cm/day.

For the determination of the *C. graminicola* growth rate, defined mycelial plugs of CgM2, CgM2::Arp1-TagRFP-T, and CgM2::SmArp1-mNG from pre-cultures were transferred to fresh CM plates without selection. After 3 d, the growth area was recorded using an Epson Perfection V600 Photo scanner in four subsequent days. The optical evaluation of the growth area was done using the measuring tool of Fiji [71] on scaled images. The growth rate of four replicates was determined by the difference of the growth area of two subsequent days in cm^2^.

## 3. Results

### 3.1. Arp1 Deletion in S. macrospora Results in a Severe Growth Defect That Can Only Partially Be Complemented

As part of the dynactin protein complex, Arp1 is highly conserved among eukaryotes. As the performed multiple sequence alignment revealed, the Arp1 protein showed a high amino-acid identify among the clades of Ascomycota, Basidiomycota, and Mucormycota in saprophytic and pathogenic species (Table 2 and Figures S1 and S2). To characterize Arp1 in *S. macrospora*, the ∆Smarp1 deletion strain was generated by the homologous recombination of an hph-deletion cassette flanked by upstream and downstream regions of Smarp1 (Figure S3a). The resulting ∆Smarp1 strain was verified via PCR (Figure S3b) and Southern blot analysis (Figure S3c). To investigate the sexual development of the deletion strain, together with the complementation strains compared to the wt *S. macrospora*, the growth behavior was analyzed over 10 d (Figure S4a). The life cycle of wt *S. macrospora* starts with a germinating ascospore that differentiates into a vegetative mycelium. After three more days, the female gametangia (ascogonia) are produced, and one day later, the unpigmented fruiting-body precursors (protoperithecia) are formed. These develop into melanized protoperithecia, and after self-fertilization, karyogamy, meiosis, and a post-meiotic-mitosis, they mature into pear-shaped perithecia. The developmental stages of pigmented protoperithecia and pigmented mature fruiting-bodies could be observed in the wt strains after 5–10 d (Figure S4a). In contrast, the ∆Smarp1 strain was arrested in the formation of perithecia and displayed a slow growth velocity even after 10 d of incubation (Figure S4a). This severe phenotype was only partially complemented by the ectopic integration of Smarp1 with and without a fluorescence tag, although Western Blot analyses verified the expression of SmARP1-TagRFP-T in the investigated strain (Figure S4b).

### 3.2. SmArp1 Localization Is a Dynamic Process at Growing Hyphal Tips and in Proximity to the Nucleus

**Figure 1:**
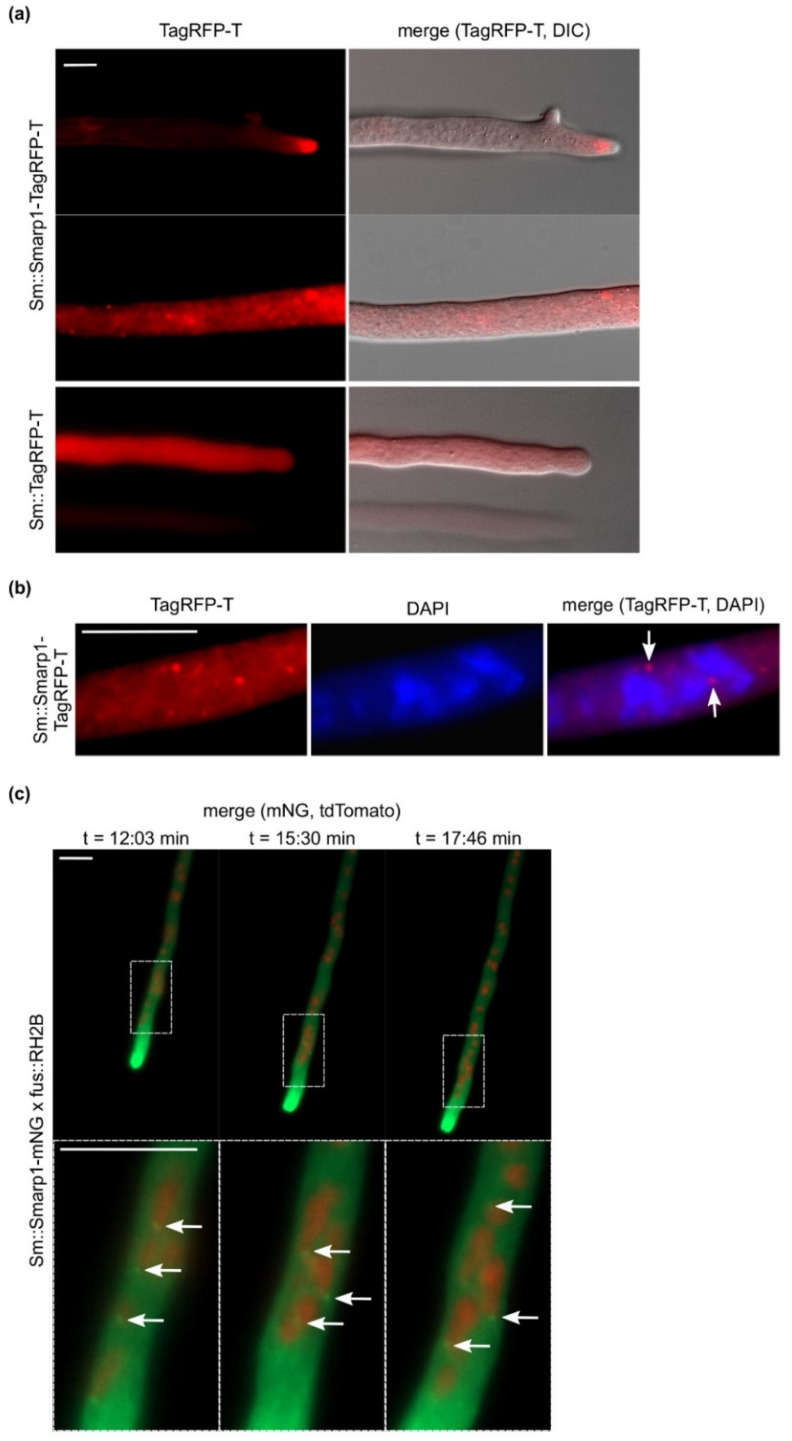
The localization of Arp1 in *S. macrospora* and the heterologous expression of pSmarp1-TagRFP-T or pSmarp1-mNG in wild-type *S. macrospora* hyphae: a recording of hyphae from growing strains on BMM-covered glass slides (**a**,**b**) or on BMM agar supplemented with agarose (**c**) after incubation for 24 h at 27 °C. (**a**) SmArp1-TagRFP-T shows subapical localization at the hyphal tip and as puncta-like structures in the hyphae. TagRFP-T expression served as the control. (**b**) The nuclear localization of SmArp1-TagRFP-T is marked by arrows. Nuclei were stained with DAPI. (**c**) Selected images of Video S2 showing the dynamic localization of SmArp1-mNG in growing hyphae after 24 h of incubation on BMM agar at 27 °C. The nuclear localization of SmArp1-mNG is indicated by arrows. Nuclei are labeled by histone 2B with tdTomato (RH2B), size bar = 10 µm.

Fluorescence microscopy was performed to determine the cellular localization of SmArp1-TagRFP-T. The fusion construct predominantly localizes as subapical structures in the tips of growing hyphae (Figure 1a). The application of a higher imaging contrast allowed for the further determination of dot-like structures apart from hyphal tips (Figure 1a). To confirm that the SmArp1 localization was independent of the used fluorophore, we replaced TagRFP-T by a C-terminal fused mNG, a monomeric green fluorescent protein derived from the lancelet *Branchiostoma lanceolatum* [59]. SmArp1-mNG localized dynamically to growing hyphal tips (Figure S5 and Video S1). To exclude probable side effects on *S. macrospora* due to the expressed fusion constructs, growth rates were determined using race tubes. No difference in growth was seen in Sm::Smarp1-TagRFP-T (3.5 ± 0.29 cm) or Sm::Smarp1-mNG (3.1 ± 0.31 cm) compared to the wild-type strain (3.4 ± 0.27 cm). The co-staining of Sm::SmArp1-TagRFP-T with DAPI (Figure 1b) and co-expression experiments with a red fluorescent histone marker (RH2B tdTomato) were performed to address the dot-like localization pattern (Figure 1c). Both experiments showed that SmArp1-TagRFP-T localized to small dots close to nuclei that moved retrograde from the tip to the inner hypha (Figure 1c and Video S2). Moreover, the evaluation of the 36 single pictures of Video S2 revealed that 6–46% of nuclei showed a close-by SmArp1-mNG localization, emphasizing that the association of Arp1 to nuclei is a highly dynamic process.

### 3.3. Dynamic Localization of Arp1 in C. graminicola

**Figure 2:**
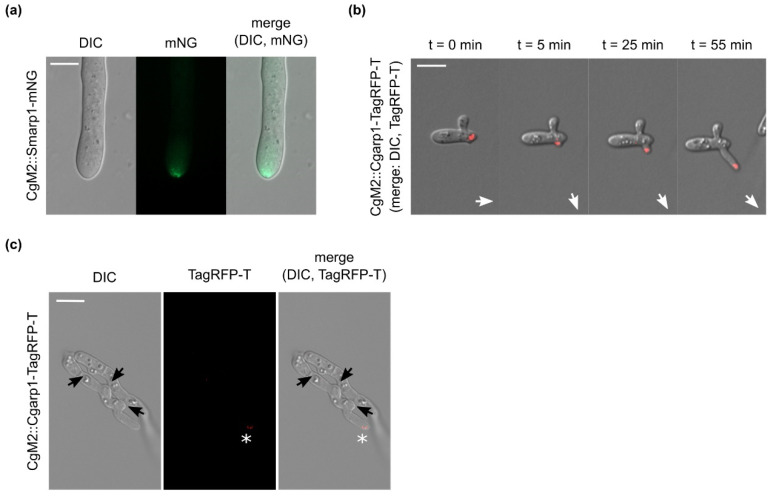
The localization of Arp1 in *C. graminicola*: (**a**) the heterologous expression of pSmarp1-mNG in wild-type *C. graminicola* CgM2 hyphae. Recording of hyphae from growing colonies on CM covered with cellophane after incubation for 3 d at 23 °C: (**b**) selected images of Video S3 showing the dynamic localization of CgArp1-TagRFP-T in growing germlings after 2 h of incubation on water agar at 23 °C. Recording interval = 5 min; polar growth axis is indicated by white arrows. (**c**) Arp1-RFP-T localization in *C. graminicola* germlings attempting for germling fusion. Germlings derived from oval conidia incubated for 6 h at 23 °C on water agar (50 µL, c = 5 × 10⁷/mL). The localization of Arp1-TagRFP-T at the tip is indicated with *. Probable future fusion sites are indicated with black arrows; size bar = 10 µm.

To follow the hypothesis that Arp1 fused to a fluorescent protein can be used as a universal marker for active growing hyphae in fungi, we chose to transform the pSmarp1-mNG plasmid into the wild-type CgM2 strain of the plant pathogen *C. graminicola*. Comparable to *S. macrospora*, the expression of Smarp1-mNG resulted in a subapical localization at the hyphal tip (Figure 2a), indicating a conserved localization among ascomycetous fungi. For the detailed investigation of localization during *C. graminicola* development and pathogenicity, CgM2 was transformed with the *C. graminicola* arp1 gene (Cgarp1) fused to TagRFP-T under the control of its native promoter. The resulting CgM2::Cgarp1-TagRFP-T strain showed a highly dynamic CgArp1 localization to growing tips of germlings. Hyphae arrested in growth completely lost tip localization (Figure 2b). At the same time, new Cgarp1-TagRFP-T patches appeared on new branching sites, following the new polar axis and indicating the current growth direction (Figure 2b and Video S3). Similar to findings for *S. macrospora*, the expression of both Arp1 fusion proteins did not result in aberrant growth patterns of *C. graminicola* transformants (CgM2: 9.9 ± 1.7 cm^2^; CgM2::Smarp1-mNG: 9.8 ± 2.2 cm^2^; CgM2::Cgarp1-TagRFP-T: 9.2 ± 1.6 cm^2^).

### 3.4. Absence of CgArp1 localization to C. graminicola future fusion points

Germling fusion is a highly dynamic process resulting in the formation of conidial anastomosis tubes (CATs) that link germinating conidia during early colony development [72]. This process includes the recognition of a probable fusion partner by a so-far unknown signal and the stepwise growth towards the counterpart in the chemotropic phase of the process [73]. Due to the observed correlation between CgArp1 localization to the polar sites of the fungal cell, we speculated about a probable localization in chemotropically active germlings during the CAT formation process. However, we were never able to monitor a clear localization of CgArp1 during germling interactions (Figure 2c), indicating a fundamental difference between growing hyphae and the fusion of CATs.

### 3.5. Arp1 Localizes Subapical to SPK

**Figure 3:**
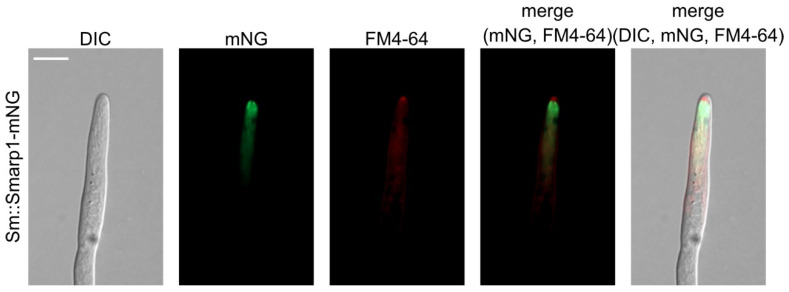
The localization of SmArp1-mNG together with FM4-64-stained hyphae in *S. macrospora*. The expression of pSmarp1-mNG in wild-type *S. macrospora* hyphae stained with FM4-64 (1 µg/mL in distilled water) and incubated for 15 min at 37 °C. The recording of hyphae was performed after growing on solid agarose-BMM for 24 h at 27 °C under continuous light conditions; scale bar = 10 µm.

The SPK, a highly organized structure at the hyphal apex that is mostly composed of secretory vesicles, is broadly used to determine active hyphal growth, growth direction, germination, and branching site selection [28,74,75,76,77]. To compare the localization of Arp1 and the SPK, we stained *S. macrospora* strains expressing SmArp1-mNG with the red fluorescent, membrane-selective dye FM4-64 [78,79]. As depicted in Figure 3, staining with FM4-64 predominantly visualized the SPK (a dense structure at the hyphal apex) and, to a lesser extent, intracellular membranes. The overlay with SmArp1-mNG revealed that Arp1 localizes just behind the SPK, verifying its subapical localization. An analogous experiment with CgM2::Smarp1-mNG showed the same cellular localization (Figure S6).

### 3.6. S. macrospora Arp1 Shows Dynamic Localization during Ascospore Germination

Based on the localization of Arp1 in actively growing hyphae, its association with the nucleus, and its subapical localization, we assumed that it could serve as a complementing marker protein in the highly polar processes of fungal development and pathogenicity. To visualize the localization of Arp1 during the ascospore germination of *S. macrospora*, Smarp1-mNG and nuclei marked with tdTomato (RH2B) fused to histone 2B were recorded in a wild-type background (Video S4). After meiosis and a post-meiotic mitosis, the asci of *S. macrospora* were found to contain eight nuclei that were surrounded by the spore wall. Subsequently, iterate mitoses led to multiple nuclei in each ascospore. During germination, spores form one germination vesicle at one side of the ascospore. While the size of the germination vesicles increases, multiple nuclei of the ascospore are released to the vesicle. Finally, hyphae start growing form the germination vesicle [80].

**Figure 4:**
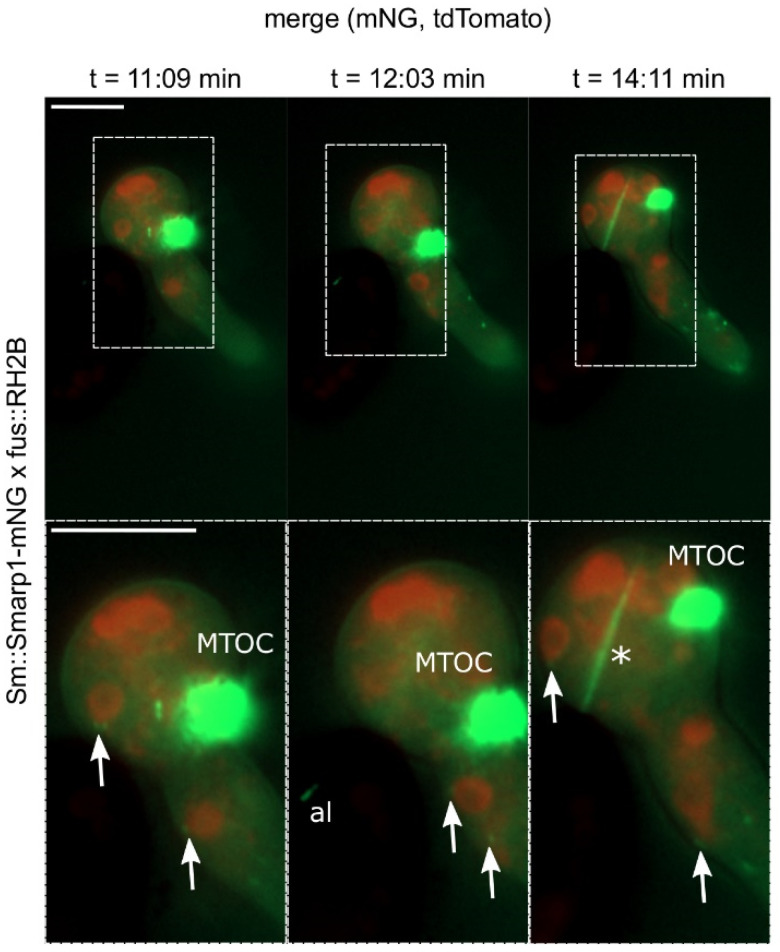
The dynamic localization of SmArp1-mNG in germinating *S. macrospora* ascospores. Selected images of Video S4, which tracks the germination of *S. macrospora* ascospores expressing *Smarp1-mNG* and red fluorescent histone 2B (RH2B tdTomato) on BMM-agar supplemented with agarose after incubation for 3–4 h at 27 °C. Arrows = localization of SmArp1-mNG close to nuclei; MTOC= putative microtubule organization center; al = SmArp1-mNG localization inside the ascospore; asterisk = SmArp1-mNG localization in fast appearing and dispersing linear structures; size bars = 10 µm; recording interval = 5 s.

During this highly dynamic germination process, we tracked SmArp1-mNG localization to four different sites and structures of the germination vesicle and the emerging hypha (Figure 4). First, Arp1 fluorescent signals appeared close to nuclei. Moreover, SmArp1-mNG localized at the growing hyphal tip, moving as dot-like structures in anterograde and retrograde directions. Third, a quickly emerging and disseminating fluorescent filamentous signal appeared in the germination vesicle. Additionally, a probable microtubule organization center (MTOC) was found to dominate the germination vesicle.

### 3.7. Analysis of Chemotropic Growth Is Improved by the Usage of the Arp1-TagRFP-T Marker

Chemotropic growth to nutrient sources, mating partners, and hosts is a fundamental process in fungi [6]. However, the assessment of the chemoattractive signal as well as cellular factors involved in signal recognition is not trivial. Recently, we established a new, easily applicable method using a 3D-printed device based on the chemotropic assay developed by Turrà et al., 2015 [69,81]. In our approach, growing *C. graminicola* germlings are confronted with a signal gradient that can be sensed over time, resulting in growth re-direction. Using a hyphal tip projection angle to a theoretical vertical line, whether a germling is growing towards the signal gradient (1–179°), is repelled by it (181–359°), or shows a neutral orientation (0°/360° and 180° [69]) can be determined. The application of this method results in *C. graminicola* germlings with a strong chemotropic growth rate when 50 mM of glucose is used as chemoattractant, whereas the growth direction of germlings is uniform in control experiments with water, as depicted by a chemotropic rate of about 50% [69].

**Figure 5:**
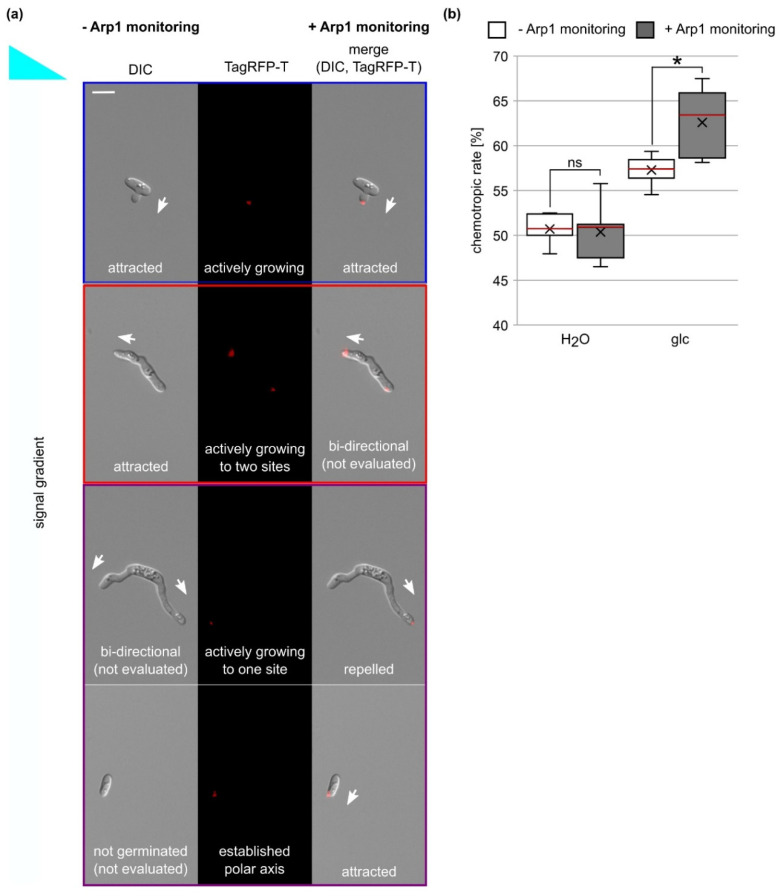
Enhanced evaluation of *C. graminicola* chemotropic growth by the application of the Arp1 marker: (**a**) the evaluation of growth direction of germlings based on microscopic analyses without (−) and with (+) Arp1 monitoring. The tip localization of Arp1-TagRFP-T was used as an indication for active growth or established polar axis. Blue box = same evaluation of results in (−) and (+); red box = different evaluation results in (−) and (+); violet box = germlings or non-germinated conidia that can be included in the (+) evaluation in contrast to (−); size bar = 10 µm. (**b**) The chemotropic rates of germlings to gradients of water (H_2_O) and 50 mM glucose (glc) after incubation for 6 h at 23 °C obtained from experiments without (−) and with (+) the monitoring of the fluorescent Arp1 marker. Chemotropic rate: <50% = repulsion; = 50% = no growth reaction; >50% = attraction; meridian is indicated in red; average is indicated by x; *n* ≥ 5; *, *p* < 0.05; ns = not significant.

Despite its significance, this evaluation mode does not differentiate between germlings showing active growth re-direction due to signal sensing and germlings that stopped their growth during incubation time and are not actively sensing. Furthermore, in these experiments, we often observed germlings that grew bi-directionally, which could not be included in the evaluation since a clear readout was missing (Figure 5a). 

Due to correlation between Arp1 tip localization and active growth or polarity axis establishment, we tested whether the usage of the Arp1 localization marker allowed us to improve the readout quality of the chemotropic assay. As depicted in Figure 5a, the localization of CgArp1-TagRFP-T to the tip often, but not always, corresponded to growth direction. In the case of bi-directional growing germlings, it allowed us to determine the current growth direction solely indicated by CgArp1-TagRFP-T localization to one hyphal tip. Interestingly, we also found that non-germinating conidia displayed a clear CgArp1-RFP-T localization to one cellular site or displayed a rather scattered localization of Arp1. Anticipating the clear targeted localization to mark the current polar axis, we were also able to include those conidia into the evaluation (Figure 5a and Figure S7, and Videos S5 and S6). To test whether the integration of the CgArp1-TagRFP-T marker changed the determined chemotropic rate, we used this strain in experiments with a 50 mM glucose gradient and experiments with no-signal control (Figure 5b). For five replicates in which at least 40 germlings were evaluated for their dominant growth direction, we monitored DIC and TagRFP-T channel information and saved them for further analysis. In a first evaluation, we referred to the information obtained from DIC channel and analyzed chemotropic growth as described earlier [69]. This quantification resulted in chemotropic rates of 50.7% ± 1.7% (water control) and 57.3 % ± 1.6 % (50 mM glucose). In a second step, we used the same microscopic pictures for a renewed evaluation, and we integrated the information gained from the Arp1 monitoring. The exclusive integration of germlings or conidia with a clear CgArp1-TagRFP-T signal in the tip or a distinct cellular site resulted in a significant increase of the chemotropic rate to 50 mM glucose (62.6% ± 3.7%; *p* = 0.01), whereas no significant changes were observed in the control experiments (50.4% ± 3.3%; *p* = 0.86). From these results, we conclude that application of the Arp1 marker can be used to increase sensitivity to analyses of chemotropic growth.

### 3.8. Tracking of Penetration Hyphae into Plant Tissue by Monitoring of Arp1 Localization in C. graminicola

**Figure 6:**
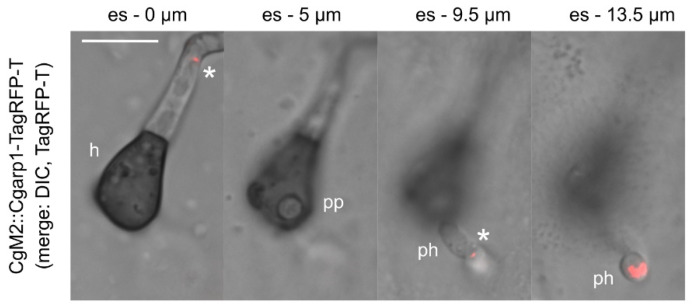
The visualization of penetration hyphae dynamics during onion epidermis perforation: 10^3^ oval conidia of *C. graminicola* CgM2::Cgarp1-TagRFP-T were inoculated on a heat-inactivated onion epidermis overlaying water agar for 29 h at 23 °C. Different layers were recorded at a fixed distance of 0.5 µm originating from the epidermal surface (es). In the selected images of Video S7, size bar = 10 µm; h = hyphopodium; pp = penetration pore; ph = penetration hyphae; * non-tip localization of Arp1-TagRFP-T.

For plant pathogens such as *C. graminicola*, the penetration of plant tissue by appressoria emerging from conidia or hyphae-derived hyphopodia marks an early step of infection. Both appressoria and hyphopodia are specialized structures that are able to build up high turgor pressure [10]. Just prior to the penetration of a plant cell, the polarity of the penetration structure shifts to a predetermined breaking point. There, a penetration pore opens and the penetration peg emerges. In a second phase, the peg develops into a penetration hypha that pushes deeper into the plant tissue [9,82]. Since a distinct signaling network regulates penetration hyphae elongation [83,84], the tracking of its path into plant tissue might help to gain important insights about the role of certain molecular players. To test whether the Arp1 marker enables such an approach, we incubated oval conidia of CgM2::Cgarp1-TagRPF-T on a heat-inactivated onion epidermis. Using stack imaging, we were able to follow the penetration hyphae from its site of emergence into plant tissue. In addition to some dot-like, dynamic CgArp1-TagRFP-T localization patches, we observed a bright fluorescent signal at the subapical tip region, indicating the current growth direction (Figure 6 and Video S7).

## 4. Discussion

In most fungi, MTs are organized by the nuclear membrane-embedded spindle pole body (SPB), also referred to as the MTOC, and found as part of intranuclear spindles and tracks within the cytoplasm [85,86]. Consequently, we monitored fluorescently labelled dynactin complex component Arp1 at distinct sites in the filaments of germlings and mature hyphae of two ascomycetous fungi, *S. macrospora* and *C. graminicola*. Highly prominent and independent of the used fluorophore, a dynamic localization of Arp1 to the subapical part of the hyphal tip was found to be correlated with an active growth of hyphae (Figure 1 and Figure 2). A similar localization was reported for *A. nidulans* expressing a GFP-tagged version of the dynein heavy chain *nudA* [87]. Additionally in axons and migrating fibroblasts during wound healing, Arp1 is enriched in the leading edge of polar growing cells [88,89]. Since the accumulation of dynactin and polarity establishment in axons and fibroblasts are simultaneous processes, it has been proposed that the dynein/dynactin complex is involved in microtubule orientation and transport rather than being a passive motor running along microtubule rails [90]. Additionally, in the corn-smut fungus *Ustilago maydis*, such a dependence of microtubule motility on the motor dynein was proposed [91], indicating a conservation of this interaction.

This hypothesis is consistent with the observed drastic reduced growth in ΔSmarp1, a phenotype previously reported for ∆ro-4, an arp1 deletion mutant generated in *N. crassa* (Figure S4) [92]. Additionally, our Arp1 localization studies in the conidia and germlings of *C. graminicola* support a role for Arp1 in transport along MTs, as well as an active role in MT organization (Figure 2). In *C. graminicola*, we observed a shift of Arp1-TagRFP-T accumulation to a new branching site before actual growth had begun.

Likewise, in the use of the Arp1-TagRPF-T marker in chemotropic growth analyses, we observed that several conidia that had not yet been germinated showed a strong and distinct localization of Arp1 to one distinct cellular site, different to dormant conidia with a uniform Arp1 localization (Figure 5 and Figure S7). Similar observations have been made for the cell end marker tea from *A. nidulans*, which appears at the future germination site prior to germ tube formation. *Tea* deletion mutant analysis showed a rather uncontrolled germination pattern at multiple sites, indicating a misfunction in polarity establishment and microtubule organization [93]. Direct evidence for dynein and dynactin in microtubule organization in filamentous fungi was derived from a study by Riquelme and co-workers (2000) in which functional dynein or dynactin complexes were correlated with fast hyphal expansion, stable polar growth, and SPK assembly [94]. To characterize the localization of Arp1 in regard to the SPK, we stained hyphae of *S. macrospora* and *C. graminicola* expressing green fluorescent Arp1 with the membrane-selective dye FM4-64. Interestingly, Arp1 was found to localize just behind the SPK in both fungi and seems to be embedded within this vesicle organization’s center. These observations are consistent with the role of dynein/dynactin in the retrograde transport of endosomal vesicles from the hyphal apex [38,41,95]. However, Arp1 and the SPK are involved in overlapping but not identical cellular functions, whereas the SPK is absent from very young germlings and correlates with germling length in *N. crassa* [96], so we also monitored fluorescent Arp1 in very young germlings of *C. graminicola* (Figure 2). Furthermore, whereas the SPK is visible at protrusions marking future cellular fusion points in *N. crassa* [97], we were not able to monitor fluorescent Arp1 at these cellular sites. This finding is in line with previous studies providing evidence that MTs are dispensable for germling communication and fusion in *N. crassa* [98]. Instead, actin patches and cables associated with endocytic vesicles and exocytosis, respectively, are enriched at germling protrusions in that fungus [98,99,100].

In addition to the importance of MTs for polarity establishment and growth, they also provide a basis for the transport of various cellular components [34]. Among others, they organize nuclear distribution along the hyphae [43,101]. In this study, we observed dynamic dot-like Arp1 patches in the mature hyphae of *S. macrospora*, which are associated with nuclei and nuclear movement in both hyphae (Figure 1) and germinating *S. macrospora* ascospores (Figure 4). Our findings are supported by an early study of Plamann and coworkers, in which *N. crassa* deletion mutants of dynein and dynactin components were investigated [43]. Phenotypes of both mutants defective in either the heavy chain of cytoplasmatic dynein (ro-1) or arp1 showed defects in polar growth, as well as evenly nuclear distribution along the fungal hyphae. Intriguingly, both deletion mutants showed a segmented accumulation of nuclei leaving other hyphal segments nuclei-free [43]. In *A. nidulans*, the coiled-coil protein ApsB is attached to the nuclei in an Arp1-like fashion during their transport along MTs and has been shown to regulate nuclear migration [101,102,103]. However, instead of localizing to the poles of the mitotic spindle, as with ApsB [102], fluorescent Arp1-mNG seemed to localize to the dynamic spindle itself in some cases, as well as to a probable MTOC in the germination vesicle (Figure 4). Though the localization of the dynein/dynactin complex to the spindle has been shown by various studies [104], such a dense Arp1 accumulation has not been monitored to date. However, one might speculate that this dense structure in the germination vesicle is potentially involved in the organized sorting of nuclei into the emerging hyphae and might be ascospore-specific. Intriguingly, this structure does not leave the vesicle, indicating that it might be a specific cellular structure required for ascospore germination. To investigate processes regarding ascospore germination in greater depth, further investigations are required.

## 5. Conclusions

Polar growth is critical for filamentous fungi and takes part in manifold processes determining fungal development and pathogenicity. Through detailed localization studies of the two filamentous fungi *S. macrospora* and *C. graminicola*, Arp1, we have provided evidence that the central component of dynactin marks sites of polarity establishment and active polar growth in filamentous fungi. Additionally, Arp1 attaches to nuclei and forms a so-far unknown structure that is probably involved in organelle sorting in germinating ascospores. Though fluorescently-tagged Arp1 shares some characteristics with established polarity markers, its localization shows some unique patterns, which makes this new marker valuable for the investigation of polar growth processes including chemotropic growth, plant penetration, and ascospore germination.

## Figures and Tables

**Table 2:** Pair-wise comparison of fungal Arp1 orthologs. Amino-acid identity is given in % from the Clustal Omega matrix of the alignment depicted in Figure S1.

	Sm	Nc	Cg	Fo	Mo	An	Ro	Ml	Cc	Sco	Cn	Um	Pc	Sp	Ca	Sc
Sm	100	100	93	92	88	78	74	74	70	73	71	74	74	51	56	50
Nc		100	92	91	88	78	74	74	70	73	71	71	74	51	56	50
Cg			100	94	87	79	74	74	69	73	71	73	76	52	57	51
Fo				100	87	78	74	74	70	74	72	74	75	51	57	50
Mo					100	78	74	75	69	70	70	73	74	51	59	52
An						100	73	74	68	72	71	71	71	50	58	51
Ro							100	99	77	81	79	81	82	54	60	50
Ml								100	77	82	78	82	81	54	60	50
Cc									100	87	80	78	74	49	54	48
Sco										100	83	82	77	52	58	51
Cn											100	80	74	52	59	50
Um												100	78	55	60	52
Pc													100	56	58	51
Sp														100	47	44
Ca															100	53
Sc																100

Abbreviations: Sm, *Sordaria macrospora*; Nc, *Neurospora crassa*; Cg, *Colletotricum graminicola*; Fo, *Fusarium oxysporum* f. sp. *Lycopersici*; Mo, *Magnaporthe oryzae*; An, *Aspergillus nidulans*; Sc, *Saccharomyces cerevisiae*; Ca, *Candida albicans*; Pc, *Pneumocystis carinii*, Sp, *Schizzosaccharomyces pombe*; Cn, *Cryptococcus neoformans* var. *grubii*; Um, *Ustilago maydis*; Cc, *Coprinopsis cinerea*; Sco, *Schizophyllum commune*; Ro, *Rhizopus oryzae*; Ml, *Mucor lusitanicus*.

## Data Availability

The data presented in this study are available on request from the corresponding author.

## Supplementary Materials

The following are available online at https://www.mdpi.com/article/10.3390/jof7070580/s1: Figure S1: CLUSTAL Omega (1.2.4) multiple sequence alignment of fungal ARP1 orthologs; Figure S2: Phylogenetic tree of Arp1 orthologs from fungi; Figure S3: Verification of *S. macrospora* *Smarp1* deletion by PCR and Southern blot analysis; Figure S4: Growth of *S. macrospora* ΔSmarp1 and complementing strains and corresponding expression controls via Western blot analysis; Figure S5: Localization of SmArp1-mNG and free mNG in *S. macrospora*; Figure S6: Localization of Arp1 and FM4-64 in *C. graminicola*. Heterologous expression of pSmarp1-mNG in *C. graminicola* wild-type CgM2 hyphae; Figure S7: Localization of Arp1 in unpolarized, non-germinated oval conidia of *C. graminicola*; Table S1: Oligonucleotides used in this study; Table S2: Plasmids used in this study; Video S1: Dynamic localization of SmArp1-mNG to growing hyphal tips; Video S2: Nuclear localization of SmArp1-mNG. Heterologous expression of *pSmarp1-mNG* in *S. macrospora* wild-type hyphae; Video S3: Dynamic localization of CgArp1-TagRFP-T in growing *C. graminicola* germlings; Video S4: Dynamic localization of SmArp1-mNG in *S. macrospora* germinating ascospores; Video S5: Establishment of a polar Arp1-TagRFP-T localization prior to germination of *C. graminicola*; Video S6: Tracking of Arp1-RFP-T during the germination of *C. graminicola* oval conidia; Video S7: Visualization of *C. graminicola* penetration hyphae dynamics during onion epidermis perforation.
