# Supplementary material for: Tracking Fungal Growth: Establishment of Arp1 as a Marker for Polarity Establishment and Active Hyphal Growth in Filamentous Ascomycetes
Source: J Fungi (Basel). 2021 Jul 20;7(7):580. doi: 10.3390/jof7070580 (PMC8304394; doi:10.3390/jof7070580)
Supplement: Supplementary file 1 [file jof-07-00580-s001.zip › supplementary files/Supplementary_Material_Grothetal_revised.pdf]

Supplementary Material

Tracking fungal growth: Establishment of dynactin Arp1 as a marker for polarity establishment and active hyphal growth in filamentous ascomycetes

Anika Groth [1], Carolin Schunke [1,2], Eva Johanna Reschka [1], Stefanie Pöggeler [1,*] and Daniela Elisabeth Nordzieke [1,*]

[1]Georg-August-University of Göttingen, Institute of Microbiology and Genetics, Department of Genetics of Eukaryotic Microorganisms, Grisebachstr. 8, 37077 Göttingen, Germany; dnordzi@gwdg.de

[2]current address: Biochemistry Center, Heidelberg University, Heidelberg, Germany

*Correspondence: spoegge@gwdg.de (S.P.), dnordzi@gwdg.de (D.E.N.)

Figures

```
Sc_ARP1        MDQLSDSYALYNQPVVIDNGSGIIK------------------AGFSGEERPKALEYCLV   42
Sp_ARP1        ---MTVTEIFDNQPICIDNGSGFIK------------------AGFAGDDIPKCLFPTCV   39
Ca_ARP1        -----MAEILYNQPVVIDNGSGNLK------------------AGFAGEDKPKSYASAII   37
Pc_ARP1        ---MEFNDVLTNQPICIDNGSGVIK------------------AGFAGEDQPKSFFPSYV   39
Um_ARP1        MAGNEFDDVLTNQPVVIDNGSGSIK------------------AGFAGQDAPKCFFPSFV   42
Ro_ARP1        -MNTEFDDVLTNQPVVIDNGSGVIK------------------AGFAGEEQPKCFFPSMV   41
Ml_Arp1        -MNSEFDDVLTNQPVVIDNGSGVIK------------------AGFAGEEQPKCFFPSMV   41
Cn_ARP1        -MATEFDDVLTNQPVVIDNGSGNIK------------------AGFAGEEQPSCYIPSFV   41
Cc_ARP1        -MSAEFDDVLTNQPVVIDNVGPLPSPGTQPTSPSSTTGIWNHKGGFCRSRSSQMLLP---   56
Sco_ARP1       -MSAEFDDVLTNQPVVIDNGSGTIK------------------AGFAGQDHPKCFFPSFV   41
An_ARP1        ----MTEATLHNAPIVIDNGSGTIR------------------AGFAGEEIPSCYFPSFV   38
Mo_ARP1        -----MAQLLHNSPIVIDNGSGTIR------------------AGFAGEDLPKCFFPSFV   37
Sm_ARP1        -----MTDSLHNAPIVLDNGSGTIR------------------AGFAGDDVPKCHFPSFV   37
Nc_ARP1        -----MTDSLHNAPIVLDNGSGTIR------------------AGFAGDDVPKCHFPSFV   37
Cg_ARP1        -----MTDSLHNAPIVLDNGSGTIR------------------AGFAGDDLPKCYFPSFV   37
Fo_ARP1        -----MADSLHNAPIVLDNGSGTIR------------------AGFAGDDLPKCYFPSWV   37
                        : * *: :** .                      .**. .   .

Sc_ARP1        GNTKYDKVMLEGL---------QGDTFIGNNA-QKLRGLLKLRYPIKHGVVEDWDSMELI   92
Sp_ARP1        GRIKHERVMPSSI---------QKDMFVGSEA-QNLRGLLKIQRPIERGIIQNWSDMEEI   89
Ca_ARP1        GRPKYQKIMAAGSTSLLSEQQSHDQLFIGNSA-QDNRGLLKLSYPIEHGIVNNWSDMEKL   96
Pc_ARP1        GRPKHLKIMAGAI---------EGDIFIGNKA-QELRGLLKIKYPIEHGIVVDWDDMERI   89
Um_ARP1        GRPKHPRVMAGAI---------EGDLFIGRKA-QELRGLLKIKYPMEHGIVTDWEDMERI   92
Ro_ARP1        GRPKHVRIMAGAV---------EGDNFIGRKA-QELRGLLKIRYPIEHGIVTDWEDMERI   91
Ml_Arp1        GRPKHVRIMAGAV---------EGDNFIGRKA-QELRGLLKIRYPIEHGIVTDWEDMERI   91
Cn_ARP1        GRPKHPRVMAGAI---------QDNLFIGRRA-QEFRGLLKIKYPMEHGVVMDWDDMERI   91
Cc_ARP1        --LFHERVMAGAL---------EGDVFIGRKA-QEFRGLLKIKYPMEHGIVTNWDDMERI   104
Sco_ARP1       GRPKHVRVMAGAL---------EGDVFIGKRA-QEFRGLLKIKYPMEHGIVTDWDDMERI   91
An_ARP1        GRPKHPRVMAGGL---------EGDVFIGQRA-QELRGLMKIRYPLEHGIVTDWDDMEKI   88
Mo_ARP1        GRPKHLKVLAGAL---------EGDVFIGEKAATELRGLLKIRYPLEHGIVTDWDDMEKI   88
Sm_ARP1        GRPKHLRVLAGAL---------EGEVFIGQKAATELRGLLKIRYPLEHGIVTDWDDMEKI   88
Nc_ARP1        GRPKHLRVLAGAL---------EGEVFIGQKAASELRGLLKIRYPLEHGIVTDWDDMEKI   88
Cg_ARP1        GRPKHLRVLAGAL---------EGEVFIGQKAATELRGLLKIRYPLEHGIVTDWDDMEKI   88
Fo_ARP1        GRPKHLRVLAGAL---------EGEVFIGQKASTELRGLLKIRYPLEHGIVTDWDDMERI   88
                   : :::  .          . : *:*  *  . ***:*:  *:::*:: :*..** :
```

```
Sc_ARP1          WSYVLNEVLQLQNIGEHPLLITEAPMNPLKNREQMAQVLFETFDVSALYVSNPAVLSLYA   152
Sp_ARP1          WSYIYSDQQLNTLPEEHPLLLTEPPLANIRNKEKIAEYFYETLNVPALSFSLQPVLALYA   149
Ca_ARP1          WYHTYTQ-DLKTQSEDHPLLITEAPLNPRSNRNKMCQILFENFNIPCIYISIQAVLSLYA   155
Pc_ARP1          WQFIYTE-ELKTVSEEHPVLLTEAPLNPRTNRDQAAQVFFETFNVPALFTSIQAVLSLYA   148
Um_ARP1          WNHVYSD-ELETLSEEHPVLLTEAPLNPRSNRDMAAQIFFETFNVPALFTSIQAVLSLYS   151
Ro_ARP1          WQYIYTD-ELKTLSEEHPVLLTEAPLNPRANRDTAAQIFFETFNVPALFTSIQAVLSLYS   150
Ml_Arp1          WQYIYTD-ELKTLSEEHPVLLTEAPLNPRANRDTAAQIFFETFNVPALFTSIQAVLSLYS   150
Cn_ARP1          WGWVYGE-GLKALSEEHPVLLTEAPLNPRQNRDIAAQIFFETFNVPAFFTSVQAVLSLYS   150
Cc_ARP1          WNWVYAE-ELGTLSEEHPVLLTEAPLNPRTNRDVAAQIFFDTFNVPALFISVQAVLSLYS   163
Sco_ARP1         WSWVYAE-ELGTLSEEHPVLLTEAPLNPRSNRDIAAQIFFDTFNVPAMFTSVQAVLSLYS   150
An_ARP1          WHYVYEN-ELKTLPEEHPVLLTEPPLNPRKNRDIAAQIMFETFNVPALYTSIQAVLSLYA   147
Mo_ARP1          WGYVYEE-GLKVLSEEHPVLLTEPPLNPRANRDTAAQILFETFNVPALYTSIQAVLSLYA   147
Sm_ARP1          WAYVYDE-GLKTLSEEHPVLLTEPPLNPRANRDTAAQILFETFNVPALYTSIQAVLSLYA   147
Nc_ARP1          WAYVYDE-GLKTLSEEHPVLLTEPPLNPRANRDTAAQILFETFNVPALYTSIQAVLSLYA   147
Cg_ARP1          WEYVYGE-GLKTLSEEHPVLLTEPPLNPRSNRDTAAQILFETFNVPALYTSIQAVLSLYA   147
Fo_ARP1          WEYVYGE-GLKTLSEEHPVLLTEPPLNPRSNRDTAAQILFETFNVPALHTSIQAVLSLYA   147
                 *     :        :**:*:** *:    *::  .: :::.::: .:  *   **:**:

Sc_ARP1          SGRTTGCVVDCGEGYCSTVPIYDGFALPASMMRMDIGG--------ADITEQLQFQLRKS   204
Sp_ARP1          SARTTGIVLECGDGLTHSVPIYDGFSIPSAIQQEEIGG--------RDVTDYLQLQLR-K   200
Ca_ARP1          SGRTTGVVIDSGDGVTHVVPVYEGFALPPSIKRMDIAG--------RDITESLAFNIRRM   207
Pc_ARP1          SGRTTGVVLDSGDGVTHAVPIYEGFAMPSAIRRIDIAG--------RDVTEYLQLLLR-K   199
Um_ARP1          SGRTTGIVLDSGDGVTHAVPVYEGFSMPNAIRRVDVAG--------RDVTENLQTHLR-K   202
Ro_ARP1          SGRTTGIVLDSGDGVTHAVPVYEGFAIPHAIRRVDIAG--------RDVTEYLQLLLR-R   201
Ml_Arp1          SGRTTGIVLDSGDGVTHAVPVYEGFAIPHAIRRVDVAG--------RDVTEYLQLLLR-K   201
Cn_ARP1          SGRTTGIVLDSGDGVTHAVPVFEGFSMPHAVRRIDLAG--------RDITDHLQLLLR-K   201
Cc_ARP1          SGRTTGIVLDSGDGVTHAVPVFEGFSMSHAIRRVDVAGSSEHGPFSRDVTDHLQLLLR-K   222
Sco_ARP1         SGRTTGIVLDSGDGVTHAVPVFEGFSMPHAIRRVDVAG--------RDVTDHLQLLLR-K   201
An_ARP1          SGRTTGVVLDSGDGVSHAVPVFEGFAIPNSIRRIDVAG--------RDVTEQLQLLLR-K   198
Mo_ARP1          SGRTTGIVLDSGDGVSHAVPVYEGFAITNSIQRIDVAG--------RDVTEHLQTLLR-K   198
Sm_ARP1          SGRTTGVVLDSGDGVSHAVPVYQGFTVPNSIRRIDVAG--------RDVTEYLQTLLR-K   198
Nc_ARP1          SGRTTGVVLDSGDGVSHAVPVYQGFTVPNSIRRIDVAG--------RDVTEYLQTLLR-K   198
Cg_ARP1          SGRTTGIVLDSGDGVSHAVPVYEGFAMPSSIRRIDVAG--------RDVTEYMQMLLR-K   198
Fo_ARP1          SGRTTGIVLDSGDGVSHAVPVYEGFAMPSSIRRIDVAG--------RDVTEYMQTLLR-K   198
                 *.**** *::.*:*    **:::**::  :: : ::.*         *:*: :   :*

Sc_ARP1          AGVSLFSSSEREIVRTMKEKVCYLAKNIKKEEEKYLQGTQ------DLISTFKLPDGRCI   258
Sp_ARP1          SGHELVSSAEKEIVREIKEKCCYVASDFRSEIESWTEHK-------PQIHTYQLPDNQTI   253
Ca_ARP1          TGVALQSSSELEIVRLIKEQNCFISKDPVRDEKKYGSHYSRNNPNNELMSTYKLPDGHEI   267
Pc_ARP1          SGTIFHTSAEKEIVRIIKEKCSYVTLDPRKEEKEWINASISGGKDYTKEEEFKLPDGNVL   259
Um_ARP1          AGYHLHTSAEKELVRIIKEKTCYVALNPTKEEKDTP-----------QHEEFKLPDGNTI   251
Ro_ARP1          SGYNFHTTAEKEVVRIIKEKTCYIALNPVKEEKETS----------GKVDDFMLPDGNII   251
Ml_Arp1          SGYNFHTTAEKEVVRIIKEKTCYIALNPAKEEKETS----------GKVDDFMLPDGNII   251
Cn_ARP1          AGHNLHTSAEKEVVRTIKEKTCYLALNPVKEEKDQG----------GAWEEFRLPDGKVI   251
Cc_ARP1          AGHHLHTSAEREVVRTIKEKCCYVALNPIKEEKDSL----------GRTEEFRLPDGNIV   272
Sco_ARP1         AGHHLHTSAEREVVRTIKEKCCYIPISPAKEEKDTN----------GRTEDFRLPDGNVV   251
An_ARP1          TGHVLHTSAEKEVVRMIKEKVCYVSLDPKREEKEWMNSYKSES----KHADYVLPDGHKI   254
Mo_ARP1          SGYVFHTSAEKEVVRLIKEATSYVAHDPKKEEKEWAGAKLDPSK---MMSDYTLPDGNKI   255
Sm_ARP1          SGYVFHTSAEKEVVRLIKESVTYVAHDPRKEEKEWAAAKMDPAK---I-AEYVLPDGNKL   254
Nc_ARP1          SGYVFHTSAEKEVVRLIKESVTYVAHDPRKEEKEWAAAKMDPAK---I-AEYVLPDGNKL   254
Cg_ARP1          SGYVFHTSAEKEVVRLIKESVTYVAHDPRKEEKEWANRT-DPTK---G-VEYILPDGHKL   253
Fo_ARP1          SGYVFHTSAEKEVVRLIKESVSYVAHDPRKEERDWVGVKPNESK---F-AEYVLPDGFKL   254
                 :*  : :::* *:** :**   ::  .   : ..                 : ***.  :

Sc_ARP1          EVGNDRYRAPEILFSPQIIGLGYDGLSDMCMQSIWKVDLDLRKPLLSSIILSGGTTTLKG   318
Sp_ARP1          TLGTECFSAPEVLFNPEMMGSEASGLHIQLFKSILLSDIDLRSTLYSNIVLSGGSTLLRG   313
Ca_ARP1          QLGVERFRATEILFNPQLIGHESPGIHELTSLAIAKTDLDLRSTLYQNIILSGGNTLLKN   327
Pc_ARP1          RLGAERFRAPEILFDPEIIGSEYSGIHQVVVDAISRVDLDLRKSLFGNIVLSGGSTLTRG   319
Um_ARP1          RLGAERFRAPEILFNPELIGQEFPGIHQVIVDSINRTDLDLRKNLFSNIVLSGGTTLTKG   311
Ro_ARP1          KLGAERFRAPEILFQPELIGEEYPGIHQVIVDCISRADLDLRKSLYSNVVLSGGSTLCKG   311
Ml_Arp1          KLGAERFRAPEILFQPELIGEEYPGIHQVIVDCIGRADLDLRKSLYSNVVLSGGSTLCKG   311
Cn_ARP1          QLGTERFLAPEILFNPELVGQEYPGVHQVIVDSINRTDLDLRKSLFSNIVLSGGSTLCTG   311
Cc_ARP1          QLGAERFRAPEILFDPELIGQEYAGVHQVVVDSINRVDLDLRKSLFSNIVLSGGSTLCRG   332
Sco_ARP1         QLGPERFRAPEILFNPEIIGQEYAGVHQVIVDAITRVDLDLRKSLYSNIVLSGGSTLTKG   311
An_ARP1          KIGQERFRAPEILFDPELIGLEYPGVHQIVQDAIIRTDLDLRKSLYLNIVLSGGSTLCKN   314
Mo_ARP1          KLGAERFRAPEILFDPEIIGLEYPGVHQMVINAIGKTDLDLRKSLYSNIVLSGGSTLTKG   315
Sm_ARP1          KIGAERFRAPEILFDPEIIGLEYPGVHQIVVDSINRTDLDLRRDLYSNIVLSGGSTLTKG   314
Nc_ARP1          KIGAERFRAPEILFDPEIIGLEYPGVHQIVVDSINRTDLDLRRDLYSNIVLSGGSTLTKG   314
Cg_ARP1          KIGAERFRAPEILFDPEIIGLEYPGVHQIVVDAINRTDLDLRKSLYSNIVLSGGSTLTRG   313
Fo_ARP1          KIGAERFRAPEILFDPEIIGLEYPGVHQIVVDAINRTDLDLRKSLYSNIVLSGGSTLTKG   314
                  :* : : * *:**.*:::*    *:      .*   *:***  *  .::****.*   .
```

```
Sc_ARP1       FGDRMLWDLEALT---------------------KGTSKIKIIAPSERKYTTWIGGSILT  357
Sp_ARP1       FGERFISELRAIS---------------------GKKNQVKIYASPERMHNAWLGGSILA  352
Ca_ARP1       FGDRMLKELKDLQQPLQEDQKMSIWNKNVQNDVYNTKMKVKIYAPPERKYSTWIGGSILA  387
Pc_ARP1       FGDRLLSEIRRLA---------------------VKDVKIKIFAPPERKYSTWIGGSILA  358
Um_ARP1       FGDRMLAEVKRLA---------------------VREMKIKIFAPPERKYATWIGGSILA  350
Ro_ARP1       FGERLLSEIKRLA---------------------LKDIKIKIYAPPERKYSTWIGGSILA  350
Ml_Arp1       FGERLLSEIKRLA---------------------LKDIKIKIYAPPERKYSTWIGGSILA  350
Cn_ARP1       FGDRLLNEVKKLA---------------------VKDVKLKIYAPPERKYSTWIGGSILA  350
Cc_ARP1       FGDRLLNEVKKLA---------------------LKDIKIKIYAPPERKYSTWIGGSILA  371
Sco_ARP1      FGDRLLNEVKKLA---------------------LKDIKIKIYAPPERKYSTWIGGSILA  350
An_ARP1       FPDRLMREIKRLA---------------------VEDMKIRISAPAERKYTTWIGGGILA  353
Mo_ARP1       FGDRLLTEVQKLA---------------------VRDMRIKIFAPPERKYSTWIGGSILA  354
Sm_ARP1       FGDRLLTEVQKLA---------------------VKDMRIKIFAPPERKYSTWIGGSILA  353
Nc_ARP1       FGDRLLTEVQKLA---------------------VKDMRIKIFAPPERKYSTWIGGSILA  353
Cg_ARP1       FGDRLLTELQRLA---------------------VKDMRIKIFAPPERKYSTWIGGSILA  352
Fo_ARP1       FGDRLLTELQKLA---------------------VKDMRIKIFAPPERKYSTWIGGSILA  353
              * :*:: ::. :                          :::* *  ** : :*:**.**:

Sc_ARP1       GLSTFQRLWTKKSDWLEDSTRVYSNLM*      384
Sp_ARP1       SLSTFRRLLITSEEYKNDQNVIFRRRF-      379
Ca_ARP1       GLSTFKKMWVTSEEYHENPDIVHVKCM-      414
Pc_ARP1       SLSTFRKMWVSAEEYQEDPDIIHRKSI-      385
Um_ARP1       GLSTFKRMWVSAEEYNEDPDVIHKKTF-      377
Ro_ARP1       SLSTFKKMWVSAEEYQEDPDIIHKKTF-      377
Ml_Arp1       SLSTFKKMWVSAEEYQEDPDIIHKKTF-      377
Cn_ARP1       GLSTFKKMWVSADEYKEDPDIIHKKAF-      377
Cc_ARP1       GLSTFKKMWVSAEEYQEDPDIIHKKFGF      399
Sco_ARP1      GLSTFKKMWVSAEEYQEDPDIIHKKTGF      378
An_ARP1       GLSTFRKMWVSADEWHEDPEIIHRKFA-      380
Mo_ARP1       GLSTFRKMWVSMDDWHENPDIIHTKFN-      381
Sm_ARP1       GLSTFRKMWVSIDDWHENPDIIHTKFT*      380
Nc_ARP1       GLSTFRKMWVSIDDWHENPDIIHTKFT-      380
Cg_ARP1       GLSTFRKMWVSIDDWHENPEIIHTKLT-      379
Fo_ARP1       GLSTFRKMWVSIDDWHENPDIIHTKFT-      380
              .****:::  . .:: ::   :. .
```

Figure S1. CLUSTAL Omega (1.2.4) multiple sequence alignment of fungal ARP1 orthologs. Accession numbers are indicated in Figure S2 (phylogenetic tree). Abbreviations: Sm, *Sordaria macrospora*; Nc, *Neurospora crassa*; Cg, *Colletotrichum graminicola*; Fo, *Fusarium oxysporum* f. sp. *Lycopersici*; Mo, *Magnaporthe oryzae*; An, *Aspergillus nidulans*; Sc, *Saccharomyces cerevisiae*; Ca, *Candida albicans*; Pc, *Pneumocystis carinii*, Sp, *Schizzosaccharomyces pombe*; Cn, *Cryptococcus neoformans* var. *grubii*; Um, *Ustilago maydis*; Cc, *Coprinopsis cinerea*; Sco, *Schizophyllum commune*; Ro, *Rhizopus oryzae*; Ml, *Mucor lusitanicus*. . In the conservation line, asterisk indicates positions, which have a single, fully conserved residue; colon indicates conservation between groups of strongly similar properties; and period indicates conservation between groups of weakly similar properties.

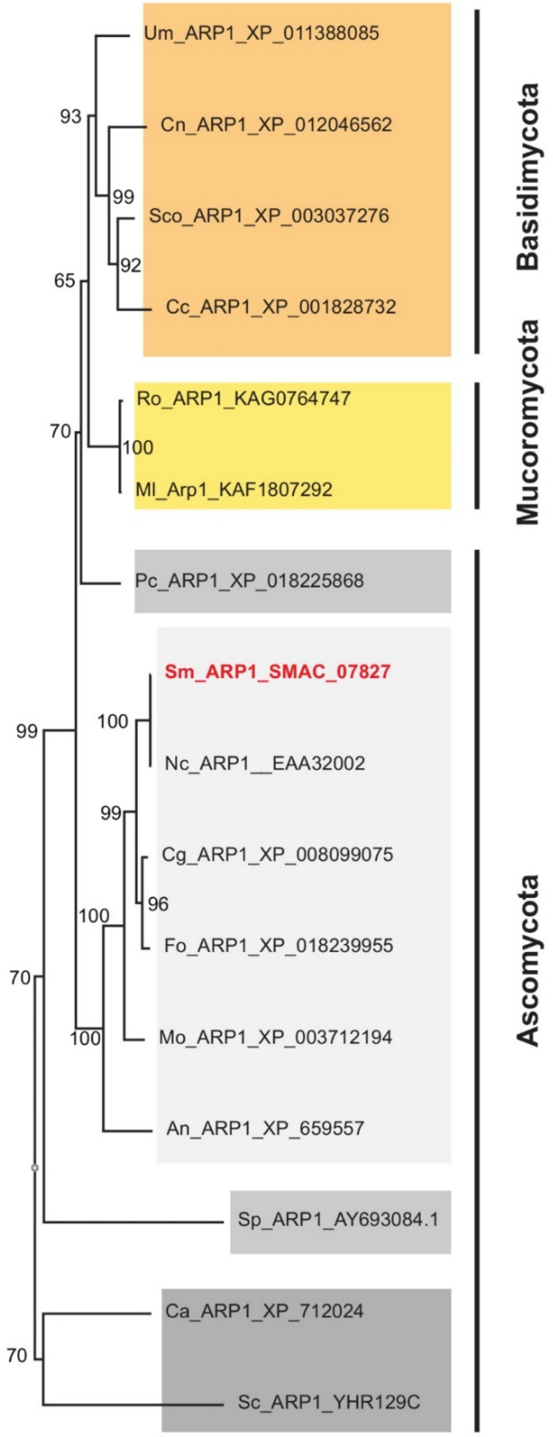

Figure S2. Phylogenetic tree of Arp1 orthologs from fungi. The phylogenetic tree of Arp1 was generated with the Neighbor Joining method. Orthologs were identified with BLASTP search using amino acid sequences of the *Sordaria macrospora* SmARP1 (SMAC_07827) indicated in red. The multiple sequence alignment and phylogenetic analysis was performed with MAFFT version 7 [1]. Bootstrap values based on 1000 replications are rounded to whole numbers and are indicated at the nodes. Accession numbers are indicated. Abbreviations: Sm, *Sordaria macrospora*; Nc, *Neurospora crassa*; Cg, *Colletotrichum graminicola*; Fo, *Fusarium oxysporum* f. sp. *Lycopersici*; Mo, *Magnaporthe oryzae*; An, *Aspergillus nidulans*; Sc, *Saccharomyces cerevisiae*; Ca, *Candida albicans*; Pc, *Pneumocystis carinii*, Sp, *Schizzosaccharomyces pombe*; Cn, *Cryptococcus neoformans* var. *grubii*; Um, *Ustilago maydis*; Cc, *Coprinopsis cinerea*; Sco, *Schizophyllum commune*; Ro, *Rhizopus oryzae*; Ml, *Mucor lusitanicus*.

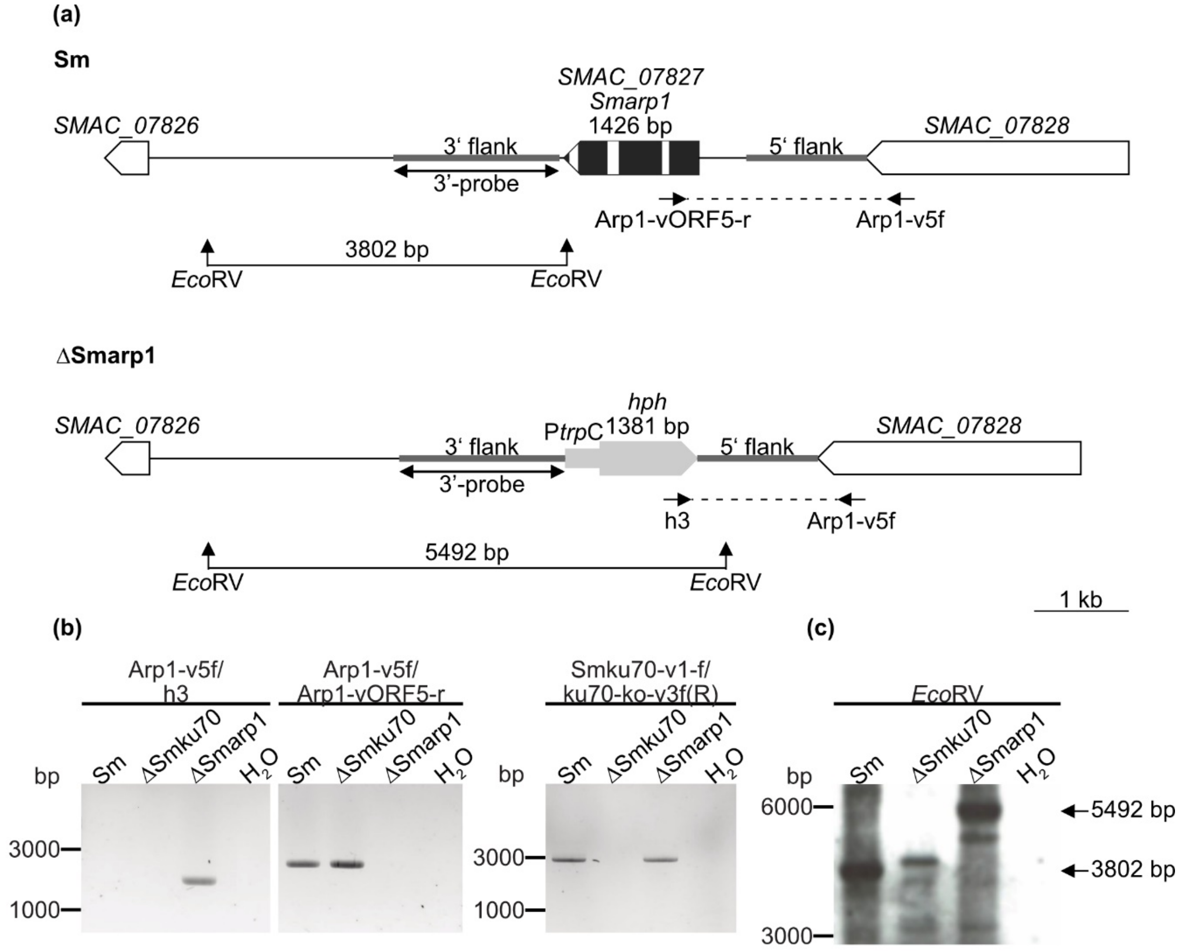

Figure S3. Verification of *S. macrospora Smarp1* deletion by PCR and Southern blot analysis. (a) Schematic representation of the Smarp1 ORF SMAC_07827 (black arrow) with three introns (white boxes). White arrows indicate adjacent ORFs. After Golden-Gate cloning, the *hph* cassette replaced the *Smarp1* ORF. Primer binding sites for the verification of *hph* cassette integration (Arp1-v5f/ h3), presence of *Smarp1* gene (Arp1-v5f/ Arp1-vORF5-r), and probe amplification for Southern hybridization are indicated. Regions of probe binding and size of the expected DNA fragments generated by *EcoR*V hydrolyzation for Sm and ΔSmarp1 are plottet. (b) PCR verification of the integration of the *hph* cassette into the *Smarp1* locus and the presence of *Smku70* gene in the *Smarp1* deletion mutant. (c) Confirmation of *Smarp1* deletion by Southern Blot analysis. Genomic DNA was hydrolyzed with *EcoR*V. Signals detected correspond to the fragment size of 3802 bp for Sm and ΔSmku70 strains and 5492 bp for the ΔSmarp1 strain (indicated by arrows).

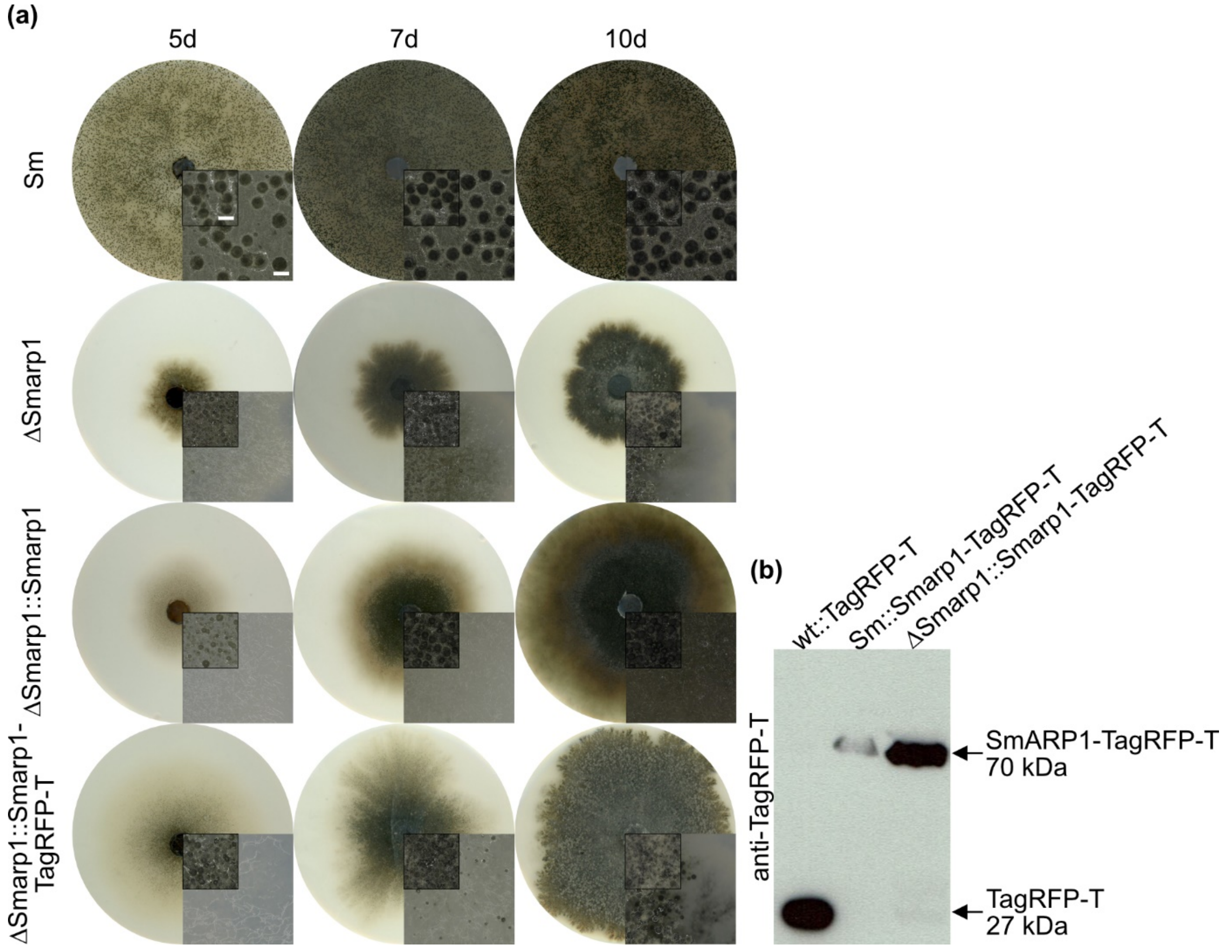

Figure S4. Growth of *S. macrospora* ΔSmarp1 and complementing strains and corresponding expression controls via Western blot analysis. (a) Phenotypic analysis of *S. macrospora* wt (Sm), ΔSmarp1 and the complementation strains ΔSmarp1::Smarp1 and ΔSmarp1::Smarp1-TagRFP-T. Strains were grown on SWG medium at 27 °C. Pictures of the agar plates and microscopic images were taken after indicated days, scale bar = 0.5 mm. (b) Western blot analysis of *S. macrospora* wt (Sm) and ΔSmarp1 ectopically expressing SmARP1-TagRFP-T, wt::TagRFP-T served as control. Expected protein sizes are indicated.

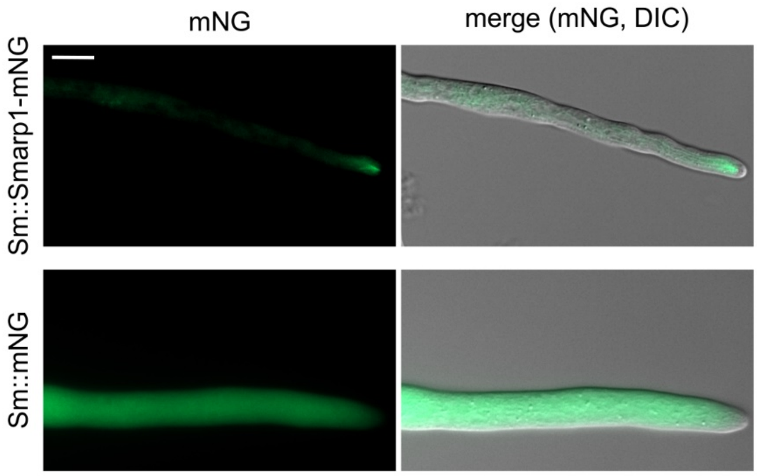

Figure S5. Localization of SmArp1-mNG and free mNG in *S. macrospora*. Heterologous expression of pSmarp1-mNG in *S. macrospora* wildtype hyphae. Recording of hyphae from growing strains on BMM-covered glass slides after incubation for 24 h at 27 °C. SmArp1-mNG shows comet-like localization at the hyphal tip. Expression of mNG serves as control, size bar = 10 µm.

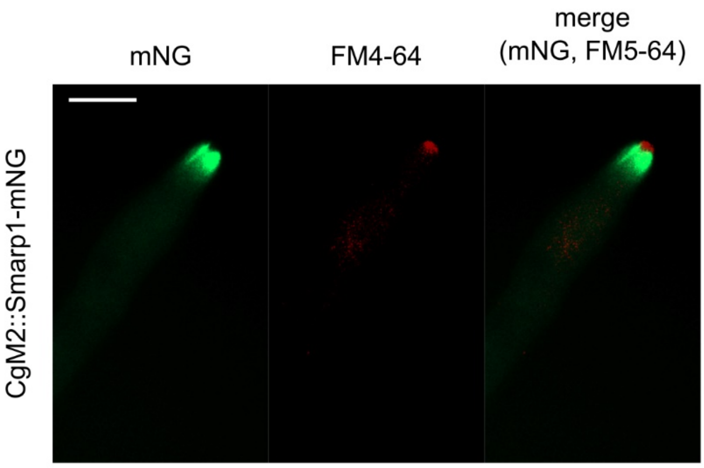

Figure S6. Localization of Arp1 and FM4-64 in *C. graminicola*. Heterologous expression of pSmarp1-mNG in *C. graminicola* wildtype CgM2 hyphae. Recording of hyphae from growing colonies on CM topped with cellophane after incubation for 3 d at 23 °C. Hyphae were stained with 1 µg/ ml FM4-64 for 15 min at 37 °C, size bar = 10 µm.

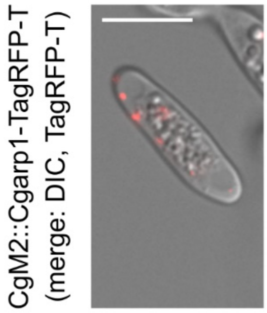

Figure S7. Localization of Arp1 in unpolarized, non-germinated oval conidia of *C. graminicola*. Oval conidia of CgM2 expressing endogenous Arp1-TagRFP-T were incubated to rate the chemotropic response to 50 mM glucose (glc) for 6 h, 23 °C in the 3D printed device, size bar = 10 µm.

Tables

Table S1. Oligonucleotides used in this study.

Oligonucleotide	Sequence (5′ to 3′)
Arp1_promotor_f	**TAAAACGACGGCCAGTGAGCGCGCGTAAT**TCAAAAATGGTTCGTGC ACACAAA
Arp1-Neo_r	**ATGTTATCCTCCTCGCCCTTGCTCACCAT**TGTAAACTTCGTATGAATG ATATCCGGA
Neo_f	ATGGTGAGCAAGGGCGAG
Neo_TtrpC_r	**GTTTGATGATTTCAGTAACGTTAAGTGGA**CTACTTGTACAGCTCGTCC ATGC
TtrpC_f	TCCACTTAACGTTACTGAAATCATCAAAC
TtrpC_pRS_r	**GCGGATAACAATTTCACACAGGAAACAGC**TCGAGTGGAGATGTGGA GTGG
Arp1_5′f	**TAAAACGACGGCCAGTGAGCGCGCGTAAT**TCAAAAATGGTTCGTGC ACA
Arp1_RFP_r	**TTAATCAGCTCTTCGCCCTTAGACACCAT**TGTAAACTTCGTATGAATG A
RFP-f	ATGGTGTCTAAGGGCGAAGAG
RFP-r-trpC	**TTTGATGATTTCAGTAACGTTAAGTGGAT**TTACTTGTACAGCTCGTCC ATGC
TrpC_F	GATCCACTTAACGTTACTGAAATCATCAAA
pRS426GFPrev	**GCGGATAACAATTTCACACAGGAAACAGC**TCGAGTGGAGATGTGGA GTG
5'CgArp1_pJet_fw	**GTTTTTCAGCAAGAT**ATCGCCAGATTTCTAGGCCCC
CgArp1_rev	CGTAAGCTTGGTGTGTATGATCT
tagRFP-T_CgArp1_fw	**CACACCAAGCTTACG**ATGGTGTCTAAGGGCGAAG
TtrpC_r	TCGAGTGGAGATGTGGAGTG
nptII_TtrpC_fw	**CACATCTCCACTCGATAT**CTCAGAAGAACTCGTCAAGAAGG
PtrpC_pJet_rev	**ATCTTCTAGAAAGAT**CTTGGCTGGAGCTAGTGGA
Arp1-v5f	TCACTTCGACCCCTTACTAAC
h3	gtactcgccgatagtggaaac
Arp1-vORF5-r	AAACACCTCGCCCTCTAATGC
Smku70-v1-f	CATCGAGGTGAGCAAGTCAATG
ku70-ko-v3f(R)	GCGCAACTCCAGCGTGACTG
GG_KO_fw	TAGGGCGAATTGGGTACCG
GG_KO_rv	GGCCGCTCTAGAACTAGTG
Arp1-ko-5f	**GACTGGTCTCA AGTC**CCCTTGGTGCAGTACATGCTC
Arp1-ko-5r	**CAGAGGTCTCA GCAG**TGGGAGGGAATGTCTTCAAGA
Arp1-ko-3f	**GTACGGTCTCG GTCA**TGATGATGATGACGACGACGA
Arp1-ko-3r	**CTCAGGTCTCC CGTA**ACGATATGAAGCTATGAAGAC
Arp1-nat-5f	**GTAACGCCAGGGTTTTCCCAGTCACGACG** CCTTGGTGCAGTACATGCTC
Arp1-nat-3r	**GCGGATAACAATTTCACACAGGAAACAGC** ACGATATGAAGCTATGAAGA

Table S2. Plasmids used in this study.

Name of plasmid	Features	Reference
pRS-nat	*amp*^{R}, *ura3*, *nat*^{R}	[2]
pRS-hyg	*amp*^{R}, *ura3*, *hyg*^{R}	[3]
p1783-1	*amp*^{R}, *ura3*, *hyg*^{R}, *Pgpd::egfp::TtrpC*	[4]
pxyl-mNG	*amp*^{R} , *ura3*, *nat*^{R}, *Pxyl::mNG::Ttrpc*	[5]
pTagRFP-T_nat	*amp*^{R}, *nat*^{R}, *Pccg1::TagRFP-T::TtrpC*	[6]
pDest-Amp	Destination vector for Golden-Gate cloning; *bla (Bsamut), lacZ* gene with two internal *Bsa*I sites: *Bsa*I(4) and *Bsa*I(7)	[7]
pGG-hph	BsaI(6)::trpC(p)::hph::BsaI(5) in pDrive; bla, kan	[7]
pArp1-KO	*amp*^{R}, *5′-flanking region and 3′-flanking region of Smarp1 interrupted by hyg*^{R} *in pDest-Amp*	this study
pSmarp1	*amp*^{R}, *ura3*, *nat*^{R}, *Smarp1P::Smarp1::Smarp1T*	this study
pSmArp1-TagRFP-T_nat	*amp*^{R}, *ura3*, *nat*^{R}, *Smarp1P::Smarp1::TagRFP-T::TtrpC*	this study
pSmArp1-TagRFP-T_hyg	*amp*^{R}, *ura3*, *hyg*^{R}, *Smarp1P::Smarp1:: TagRFP-T::TtrpC*	this study
pSmArp1-mNG	*amp*^{R}, *ura3*, *nat*^{R}, *Smarp1P::Smarp1::mNG::TtrpC*	this study
pII99	*5.3-kb nptII, amp*^{R}	[8]
pJet1.2	*amp*^{R}	ThermoFisher Scientific
pCgArp1-TagRFP-T	*amp*^{R}, *gen*^{R}, *Cgarp1P::Cgarp1::TagRFP-T::TtrpC*	this study
pJet_gen	*amp*^{R}, *gen*^{R}	this study

nat^R: nourseothricin resistant, *hyg*^{R}: hygromycin resistant; *gen*^{R}: geneticin disulphate resistant; *amp*^{R}: ampicillin resistance; *kan*^{R}: kanamycin resistance, *ura3*: Orotidine-5'-phosphate decarboxylase gene of *S. cerevisiae*; *Pgpd*: promoter of the glycerinaldehyd-3-phosphat-dehydrogenase-gene of *A. nidulans*; *Pccg1*: promoter of the *clock controlled gene 1* of *N. crassa*; native promotors of genes are named 5′; *TtrpC*: terminator of the anthranilate synthase gene of *A. nidulans*; *mNG*: gene for green fluorescence protein monomeric NeonGreen (mNG) of *Branchiostoma lanceolatum*; *egfp*: gene for enhanced green fluorescent protein (eGFP) of *Aequorea Victoria*; *TagRFP-T*: gene for red fluorescence protein TagRFP-T of *Entacmaea quadricolor*, *nptII*: neomycin phosphotransferase gene

Videos

Video 1. Dynamic localization of SmArp1-mNG to growing hyphal tips. Heterologous expression of pSmarp1-mNG in *S. macrospora* wildtype hyphae. Recording of hyphae from growing strains on BMM-covered glass slides after incubation for 24 h at 27 °C, recording interval = 5 s, size bar = 10 µm.

Video 2. Nuclear localization of SmArp1-mNG. Heterologous expression of pSmarp1-mNG in *S. macrospora* wildtype hyphae. Nuclei are labeled by histone 2B with tdTomato (RH2B). Recording of hyphae from growing strains on BMM-covered glass slides after incubation for 24 h at 27 °C, recording interval = 5 s, size bar = 10 µm.

Video 3. Dynamic localization of CgArp1-TagRFP-T in growing *C. graminicola* germlings. Heterologous expression of CgArp1-TagRFP-T in *C. graminicola* wildtype CgM2. Tracking of Arp1-TagRFP-T in germlings derived from oval conidia after 2 h of incubation on water agar, 23 °C. Recording interval = 5 min, size bar = 10 µm.

Video 4. Dynamic localization of SmArp1-mNG in *S. macrospora* germinating ascospores. Germinating *S. macrospora* ascospores which express Smarp1-mNG and red fluorescent histone 2B (RH2B tdTomato) on BMM-agar supplemented with agarose after incubation for 3-4 h at 27 °C, recording interval = 5 s, size bars = 10 µm.

Video 5. Establishment of a polar Arp1-TagRFP-T localization prior to germination of *C. graminicola*. Heterologous expression of CgArp1-TagRFP-T in *C. graminicola* wildtype CgM2. Tracking of Arp1-TagRFP-T in oval conidia after 2 h of incubation on water agar, 23 °C. Recording interval = 5 min, size bar = 10 µm.

Video 6. Tracking of Arp1-RFP-T during the germination of *C. graminicola* oval conidia. Heterologous expression of CgArp1-TagRFP-T in *C. graminicola* wildtype CgM2. Tracking of Arp1-TagRFP-T in oval conidia after 2 h of incubation on water agar, 23 °C. Recording interval = 5 min, size bar = 10 µm.

Video 7. Visualization of *C. graminicola* penetration hyphae dynamics during onion epidermis perforation. 10^3 oval conidia of *C. graminicola* CgM2::Cgarp1-TagRFP-T were inoculated on heat-inactivated on-ion epidermis overlaying water agar for 29 h at 23 °C, recording interval = 0.5 µm, size bars = 10 µm.

References

1. Sievers, F.; Wilm, A.; Dineen, D.; Gibson, T.J.; Karplus, K.; Li, W.; Lopez, R.; McWilliam, H.; Remmert, M.; Söding, J. Fast, scalable generation of high-quality protein multiple sequence alignments using Clustal Omega. *Molecular systems biology* **2011**, *7*, 539.
2. Klix, V.; Nowrousian, M.; Ringelberg, C.; Loros, J.J.; Dunlap, J.C.; Pöggeler, S. Functional characterization of MAT1-1-specific mating-type genes in the homothallic ascomycete *Sordaria macrospora* provides new insights into essential and nonessential sexual regulators. *Eukaryot Cell* **2010**, *9*, 894-905, doi:10.1128/EC.00019-10.
3. Bloemendal, S.; Bernhards, Y.; Bartho, K.; Dettmann, A.; Voigt, O.; Teichert, I.; Seiler, S.; Wolters, D.A.; Pöggeler, S.; Kück, U. A homologue of the human STRIPAK complex controls sexual development in fungi. *Mol Microbiol* **2012**, *84*, 310-323, doi:10.1111/j.1365-2958.2012.08024.x.

4. Pöggeler, S.; Masloff, S.; Hoff, B.; Mayrhofer, S.; Kück, U. Versatile EGFP reporter plasmids for cellular localization of recombinant gene products in filamentous fungi. *Curr Genet* **2003**, *43*, 54-61, doi:10.1007/s00294-003-0370-y.
5. Werner, A.; Otte, K.L.; Stahlhut, G.; Pöggeler, S. Establishment of the monomeric yellow-green fluorescent protein mNeonGreen for life cell imaging in mycelial fungi. *AMB Express* **2020**, *10*, 222, doi:10.1186/s13568-020-01160-x.
6. Werner, A.; Otte, K.; Stahlhut, G.; Hanke, L.M.; Pöggeler, S. The glyoxysomal protease LON2 is involved in fruiting-body development, ascosporogenesis and stress resistance in *Sordaria macrospora*. *Journal of Fungi* **2021**, *7*, 82.
7. Dahlmann, T.A.; Terfehr, D.; Becker, K.; Teichert, I. Golden Gate vectors for efficient gene fusion and gene deletion in diverse filamentous fungi. *Current genetics* **2021**, *67*, 317-330.
8. Shibayama, M.; Ooi, K.; Johnson, R.; Scott, B.; Itoh, Y. Suppression of tandem-multimer formation during genetic transformation of the mycotoxin-producing fungus *Penicillium paxilli* by disrupting an orthologue of *Aspergillus nidulans uvsC*. *Current genetics* **2002**, *42*, 59-65.
